# Study on the optimal scheme of shale complex cracks formation based on Xsite discrete lattice method

**DOI:** 10.1371/journal.pone.0314157

**Published:** 2024-12-13

**Authors:** Haoyong Huang, Guozhou Qiu, Jianfa Wu, Yintong Guo, Shuai Cui, Zhen Zhang

**Affiliations:** 1 Shale Gas Research Institute of PetroChina Southwest Oil & Gas Field Company, Chengdu, Sichuan, China; 2 Key Laboratory of Geomechanics and Geotechnical Engineering safety, Institute of Rock and Soil Mechanics, Chinese Academy of Sciences, Wuhan, China; 3 School of Pipeline and Civil Engineering, China University of Petroleum (East China), Qingdao, China; Khalifa University of Science and Technology, UNITED ARAB EMIRATES

## Abstract

In the contemporary energy industry, shale gas, as an important unconventional energy resource, has been widely concerned. However, the exploitation of shale gas is faced with complex geological conditions and technical challenges, one of the main challenges is that it is difficult to form discrete complex crack networks in shale, which greatly reduces the recovery rate. For different geological conditions and engineering needs, the criteria for evaluating the effect of reservoir reconstruction will be different. The XSite discrete lattice method can simulate the crack development process and provide detailed crack morphology and characteristic information (crack area, crack volume, etc.). The advantage of the orthogonal experimental design scheme is that it can obtain as much information as possible in a relatively small number of tests, improving the efficiency and cost effectiveness of the test. Therefore, based on Xsite design 6 factor 5 horizontal orthogonal test, this paper obtained the optimal fracturing design scheme with crack area, crack shape volume, tensile crack area and shear crack area as evaluation criteria. The standard deviation of each influencing factor was calculated to obtain the optimal fracturing scheme under different evaluation criteria. And considering a variety of quantitative indicators, calculate the influence weight of each influencing factor, and get the optimal fracturing scheme considering a variety of evaluation basis. Two Wells with different depth and natural fracture development were selected to verify the feasibility of orthogonal simulation test by changing fracturing fluid rate. To provide scientific basis and technical support for optimizing shale gas exploitation scheme.

## 1 Introduction

Shale reservoirs are difficult to extract oil and gas efficiently under natural conditions because of their low porosity and permeability [1]. However, the construction of complex crack networks through hydraulic fracturing can significantly improve permeability and optimize oil and gas recovery efficiency and production [2, 3]. This method not only promotes the flow of oil and gas to the wellhead, reduces the difficulty and cost of exploitation [4], but is especially important for economically marginal shale oil and gas fields to promote the feasibility of economic exploitation [5]. Optimizing crack design can effectively connect the crack system, improve the stable productivity of oil and gas Wells, and extend the economic production cycle of Wells [6]. Fracturing scheme design not only improve resource efficiency, but also reduce environmental impacts, such as reducing water and chemicals, relieving pressure on surface facilities, and reducing earthquake risk [7]. The implementation of high efficiency crack formation program is of far-reaching significance for improving resource recovery rate and coping with resource shortage [8].

In shale development, the ideal crack network should be three-dimensional to maximize rock permeability and oil and gas recovery, but precisely controlling crack development in three-dimensional space is challenging and requires precision technology and complex operations [8, 9]. Hydraulic fracturing is a mainstream technology, despite the limitations of fracturing fluid selection, pressure control, and crack monitoring [10]. In laboratory studies, it is found that high viscosity and high injection rate are conducive to improving the complexity of hydraulic cracks [11], However, the high viscosity fracturing fluid will make it difficult for the reservoir rock to crack, and may lead to the blockage of the crack and affect the expansion range of the crack [12]. Although high injection rate can also improve the complexity of cracks, the expansion direction of hydraulic cracks is easy to lose control under the action of high injection rate, which increases the probability of a large amount of fracturing fluid loss [13]. Moreover, the crack initiation pressure of deep shale is large, and the upper limit of pump pressure is very high [14]. Therefore, to improve hydraulic fracturing results through injection rate, more stable platforms and more powerful equipment are needed, which greatly increases production costs [15].

For different geological environment and different engineering background, the evaluation criteria of reservoir reconstruction are different. For shallow shale, the crack width is larger because of the high ground stress [16], therefore, the total crack area should be used as the evaluation criterion [17]. The larger the total crack area is, the more reservoir space is connected by hydraulic cracks [18]. However, for deep shale, the crack propagation is difficult because of the high ground stress [19], even if the cracks expand smoothly, most of them will expand the weak plane of the rock with low strength. Especially, the existence of shale bedding is easy to greatly increase the area of forming tensile cracks. Therefore, in this case, the crack morphology volume and the area of tensile cracks cannot be a good evaluation criterion [20]. Accordingly, under this condition, the increase in the area of shear cracks indicates that the probability of cracks appearing at a certain angle to the bedding in the shale is also increasing, and the cracks no longer communicate with the bedding in a large area, so that the overall shape of the cracks presents spatial characteristics [21]. Hence, the research on the formation methods of deep shale complex cracks can provide a basis for the design of fracturing construction parameters under different engineering backgrounds.

Hydraulic fracturing test aims to improve the complexity of fracture, but there are many factors affecting the complexity of fracture, and the influence degree of different factors is different. Orthogonal experiment is a statistics-based multi-factor and multi-level experimental design method, which is mainly used to analyze the influence of multiple variables on the experimental results [22]. Its core idea is to obtain enough information to the maximum extent through reasonable design of a small number of experimental combinations, so as to reduce the number of experiments and costs [23]. Orthogonal tests use orthogonal tables to arrange the experiments to ensure that the different levels of each factor are reasonably distributed in each experiment to achieve uniform dispersion and strong comparative experimental design [24]. The influence of multiple factors on the experimental results can be studied simultaneously. Through the combination of scientific experiments, reduce the number of experiments, reduce the cost and time of experiments.

In view of the unclear formation method of complex cracks, this study aims to deeply study the simulation method of complex crack morphology in actual shale based on XSite discrete lattice method, and explore the formation mechanism and influencing factors of crack network, so as to provide scientific basis and technical support for optimizing shale gas mining schemes. The orthogonal experiment of 6 factors and 5 levels was designed to propose fracturing schemes suitable for various engineering backgrounds. And two Wells with completely different properties were selected to verify the accuracy of the simulation test.

## 2 Crack propagation criteria in Xsite

### 2.1 Crack propagation criterion based on J-integral

In the research of XSite, it focuses on the exploration of energy-based principles and the application of J-integral-based crack propagation criteria to practice [25]. Adopting a standard based on J-integration brings many advantages, the main advantage being that it is resolution-independent and can be easily applied in a system. Prior to the implementation of this scheme, several key topics were studied in depth, including the formulation of J-integrals in 3D environments, the expression of J-integrals in discrete forms, and the importance of accurate estimation of J-values [26]. The key to crack propagation is based on the relationship between the energy release rate G and the stress intensity factor K, which is a core consideration during incremental crack propagation [27].


$$G=\frac{K_{I}^{2}}{E^{\prime }}$$ (1)

Where,

*E′* = *E*, MPa(Plane stress);

*E*′ = *E*(1 − *ν*^2^), MPa(Plane strain);

*E*—Young modulus, MPa;

*K*_*I*_—Stress intensity factor, (MPa·m)^0.5.

According to crack theory, the process of crack propagation can be described by evaluating the energy release rate (G value) of the material. This critical G value is a key parameter for crack growth, which marks the critical point at which the material begins to crack growth under the action of external loads. At this stage, cracks begin to propagate inside the material, leading to damage and destruction of the material [28]. For open mode:

$$G_{IC}=\frac{K_{IC}^{2}}{E^{\prime }}$$ (2)

Where,

*K*_*IC*_—crack toughness(Plane strain), (MPa·m)^0.5.

The energy release rate can be evaluated with the help of J-integration. J-integral can measure the degree of energy concentration near the crack tip and capture the characteristics of crack growth [29]. By calculating the J-integral, the strain energy density at the crack tip can be obtained, and the energy released in the process of crack growth can be obtained. The contour integral *J* is defined as follows [25, 30]:

$$J=\int_{C}\Omega dx_{2}-\int_{C}T_{i}\frac{\partial u_{i}}{\partial x_{1}}ds$$ (3)

Where,

*x*_*i*_—Local cartesian coordinates, zero dimension;

Ω—Strain energy density, J/m^2^;

*T*_*i*_, *u*_*i*_—Traction and displacement along profile C, N, m.

In the case of a closed contour,. Furthermore, the J-integral taken along the unclosed profile between the no-load crack surfaces is independent of the path [31].generally speaking,

$$J=-\frac{\partial V}{\partial a}$$ (4)

Where,

*V*—Potential energy per unit thickness, J/m;

*a*—Crack length, m.

For the case of linear elasticity [25]:

J=G (5)

In other words, J is a measure of the energy release rate during the propagation of small cracks and is also applicable in the presence of crack tip plasticity [32]. The critical value of J for which crack growth occurs (Liu and Qu et al., 2019) is given by the following equation [33]

$$J_{IC}=G_{IC}$$ (6)

The final expression of the crack growth criterion is obtained by considering Eq (2) [34]:

$$J_{IC}=\frac{K_{IC}^{2}}{E^{\prime }}$$ (7)

### 2.2 J-integral in general form

In a more general form, the J-integral is viewed as a vector that is integrated counterclockwise along the contour C [25].


$$J_{i}=\int_{C}(Wn_{i}-t_{k}\frac{\partial u_{k}}{\partial x_{i}})ds$$ (8)

Where,

W—Strain energy density, J/m^2^;

n—Normal unit of curve C, dimensionless;

*t*_*k*_ = *σ*_*jk*_*n*_*j*_—Traction vector, dimensionless;

*σ*—Cauchy stress tensor, MPa;

u—Displacement vector, m.

The strain energy density is defined as:

$$W=\int_{0}^{\varepsilon }\sigma_{ij}d\varepsilon_{ij}$$ (9)

Where,

*ε*—Strain tensor, dimensionless.

### 2.3 Expression of J-integral in 3D

Under quasi-static conditions, it is assumed that the force *F*_*i*_ and tractive force *T*_*i*_ crack surface [25, 33]:

$$J=\frac{1}{R}\int \left[(\sigma_{ij}u_{j,k}-W\delta_{ki})q_{,i}n_{k}-F_{i}u_{i,j}qn_{j}\right]dV-\frac{1}{R}\int_{S}T_{j}u_{j,k}qn_{k}dS$$ (10)

Where,

V—The field of point P, dimensionless;

2R—Length of the front of the crack, m;

W—Strain energy density, J/m2;

q—Function in V, dimensionless.

For the application of J-integrals in three dimensions, the choice function q provides some flexibility when considering the spherical domain V of radius R, which applies to the spherical region of radius R [35]:

$$q=1-\overset{⌢}{r}\;\text{for}\;\left|\overset{⌢}{r}\right|<1\;\text{otherwise}\;q=0$$ (11)

Where,

$$\overset{⌢}{r}=\sqrt{{\overset{⌢}{x}}_{1}^{2}+{\overset{⌢}{x}}_{2}^{2}+{\overset{⌢}{x}}_{3}^{2}}$$ (12)

And,

$${\overset{⌢}{x}}_{i}=\frac{x_{i}-x_{i}^{c}}{R}$$ (13)

Where,

$x_{1}^{c},x_{2}^{c},x_{3}^{c}$—Coordinates of the center point of the sphere, dimensionless.

## 3 Model parameters setting

The numerical simulation of crack morphology was carried out based on XSite discrete lattice method. In order to accurately simulate the complex crack network in the actual shale, a series of simulation model parameters were set, as shown in Table 1, these parameters were conventional parameters, determined according to test experience and field work experience. These parameters include shale geological characteristics, crack geometry, rock mechanical properties and other important parameters, their selection and setting is very important for the accuracy and reliability of simulation results.

**Table 1:** Test model parameters.

Name of parameters	Parameter values
Dimension of model	50×50×50m
Elastic modulus of shale	15GPa
Poisson’s ratio	0.35
Rock density of Shale	2600kg/m^3^
Porosity	2%
Permeability	1×e^-13^m^2^
crack toughness	0.5MPa·m^0.5
Bedding cohesion	15MPa
Tensile strength of bedding	8MPa
Horizontal stress difference	15MPa

## 4 Orthogonal experimental scheme design

In hydraulic fracturing test, the factors that affect the effect of reservoir reconstruction are complicated. Specifically, the number of bedding and the friction Angle of bedding are important factors that determine the propagation path of cracks [6]. The number of bedding can lead to fracture deflection, branching and even stagnation, while the bedding friction Angle affects whether the fracture can penetrate the bedding [36]. In addition, the Angle of the high-angle crack is another key factor. The high Angle fracture can be used as a natural diversion channel, but its Angle determines the difficulty of fracturing fluid entering the fracture and the reopening of the fracture during fracturing. Flow rate (injection rate of fracturing fluid) and viscosity (rheological properties of fracturing fluid) are important parameters in fracturing operation, which directly affect the length and width of fracture extension [37]. The high flow rate facilitates rapid fracture propagation, while the high viscosity helps to maintain fracture openness, reduce fluid loss, and enhance fracture performance. However, high displacement and viscosity may lead to premature fracture branching and affect the final morphology of the fracture. Finally, the vertical stress difference (that is, the stress difference between different layers) has a significant effect on the vertical propagation of cracks. Therefore, in the process of orthogonal test design, systematic variable control and optimization design are carried out according to the above six main factors [38], including the number of bedding, bedding friction Angle, high-angle fracture Angle, displacement, viscosity and vertical stress difference, which can effectively explore the specific influence of each factor on hydraulic fracturing effect and provide an important experimental basis for optimizing the reservoir reconstruction scheme. Through orthogonal test, the relationship between the primary and secondary effects of various factors on hydraulic fracturing effect can be analyzed in a limited number of experiments, and the optimal combination scheme can be found to improve fracturing efficiency and reservoir reconstruction effect [39].

The L25(5^6) orthogonal experimental design with 6 factors and 5 levels was adopted to ensure the comprehensiveness and systematicness of the crack morphology simulation. This orthogonal experimental design scheme is widely used in experimental design, which can effectively reduce the number of tests and ensure the reliability and validity of test results [40]. Through systematic exploration and analysis of key factors in the process of crack simulation, we can better understand the formation mechanism of crack morphology. Factor levels are shown in Table 2:

**Table 2:** Factors and horizontal variables.

H F	Bedding number	Bedding friction Angle/°	High angle fracture dip angle/°	Injection rate/m^3^/min	Viscosity/mPa.s	σv-σh/MPa
1	1	1	0	2	5	5
2	2	15	30	3	10	7.5
3	3	30	45	4	15	10
4	4	45	75	5	20	12.5
5	5	60	90	6	25	15

The experimental design scheme is shown in Table 3.

**Table 3:** 6 factors 5 level experimental design scheme.

H F	Bedding number	Bedding friction Angle/°	High angle fracture dip angle/°	Injection rate/m^3^/min	Viscosity/mPa.s	σv-σh/MPa
Test1	1	1	0	2	5	5
Test2	1	15	30	3	10	7.5
Test3	1	30	45	4	15	10
Test4	1	45	75	5	20	12.5
Test5	1	60	90	6	25	15
Test6	2	1	30	4	20	15
Test7	2	15	45	5	25	5
Test8	2	30	75	6	5	7.5
Test9	2	45	90	2	10	10
Test10	2	60	0	3	15	12.5
Test11	3	1	45	6	10	12.5
Test12	3	15	75	2	15	15
Test13	3	30	90	3	20	5
Test14	3	45	0	4	25	7.5
Test15	3	60	30	5	5	10
Test16	4	1	75	3	25	10
Test17	4	15	90	4	5	12.5
Test18	4	30	0	5	10	15
Test19	4	45	30	6	15	5
Test20	4	60	45	2	20	10
Test21	5	1	90	5	15	7.5
Test22	5	15	0	6	20	10
Test23	5	30	30	2	25	12.5
Test24	5	45	45	3	5	15
Test25	5	60	75	4	10	5

## 5 Optimal scheme design results

### 5.1 Test results

**Fig 1:**
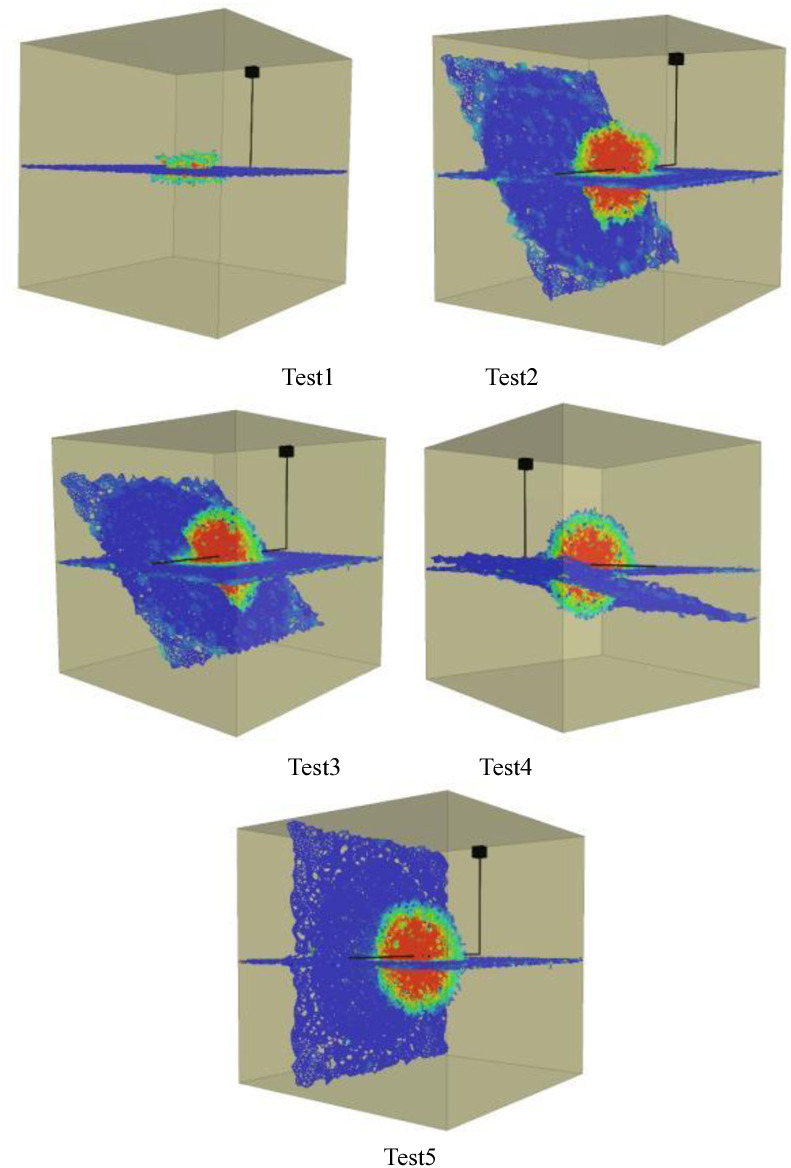
Graph of orthogonal test results(Bedding number 1). Test1, Test2, Test3, Test4, Test5.

**Fig 2:**
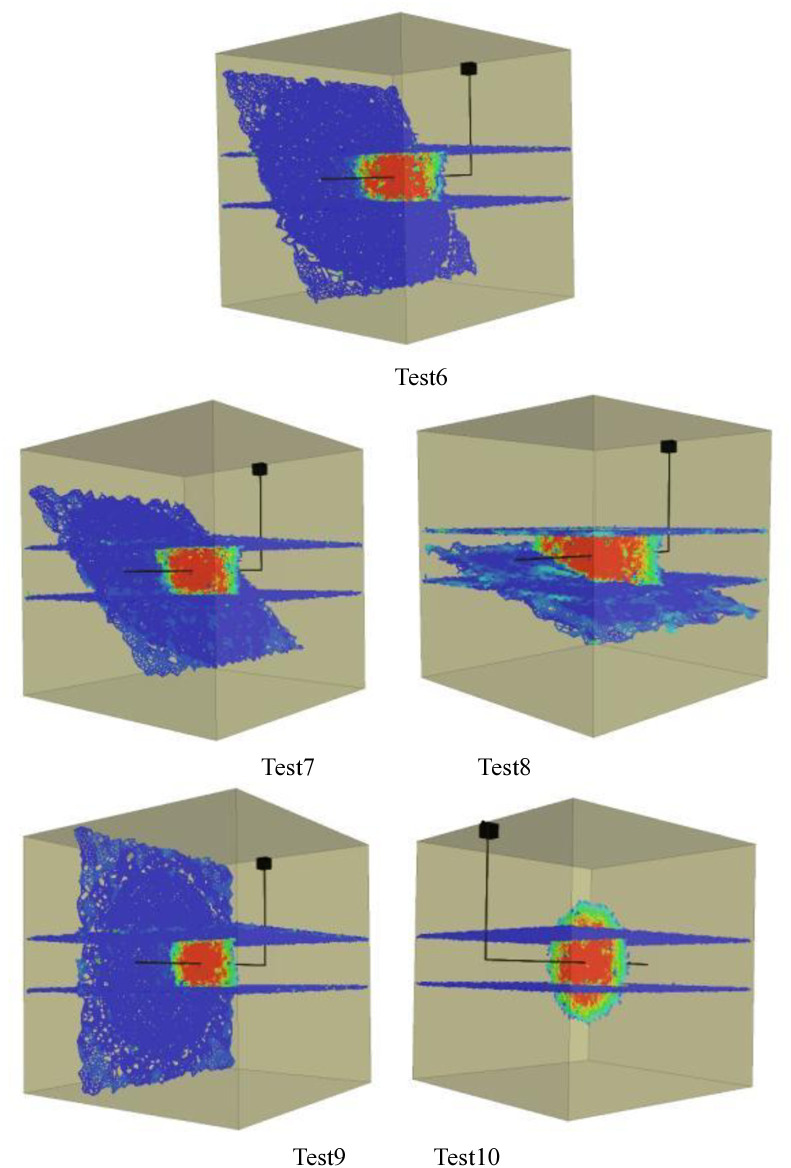
Graph of orthogonal test results(Bedding number 2). Test6, Test7, Test8, Test9, Test10.

**Fig 3:**
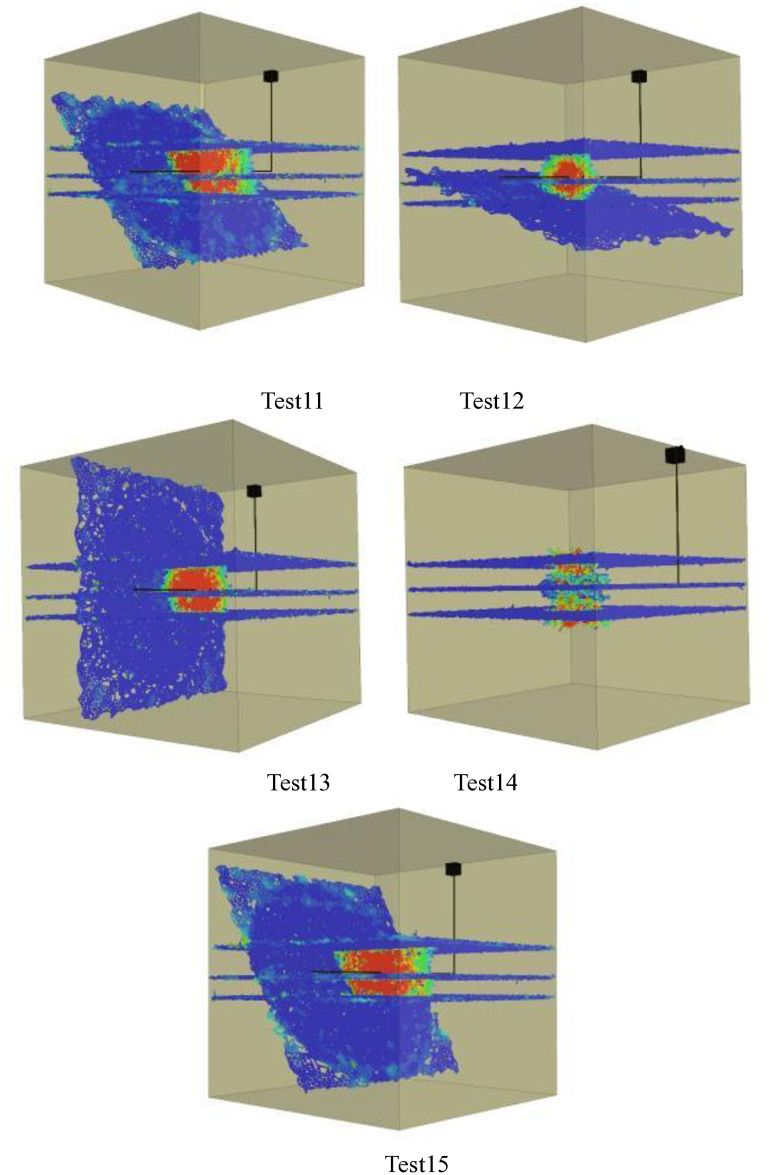
Graph of orthogonal test results(Bedding number 3). Test11, Test12, Test13, Test14, Test15.

**Fig 4:**
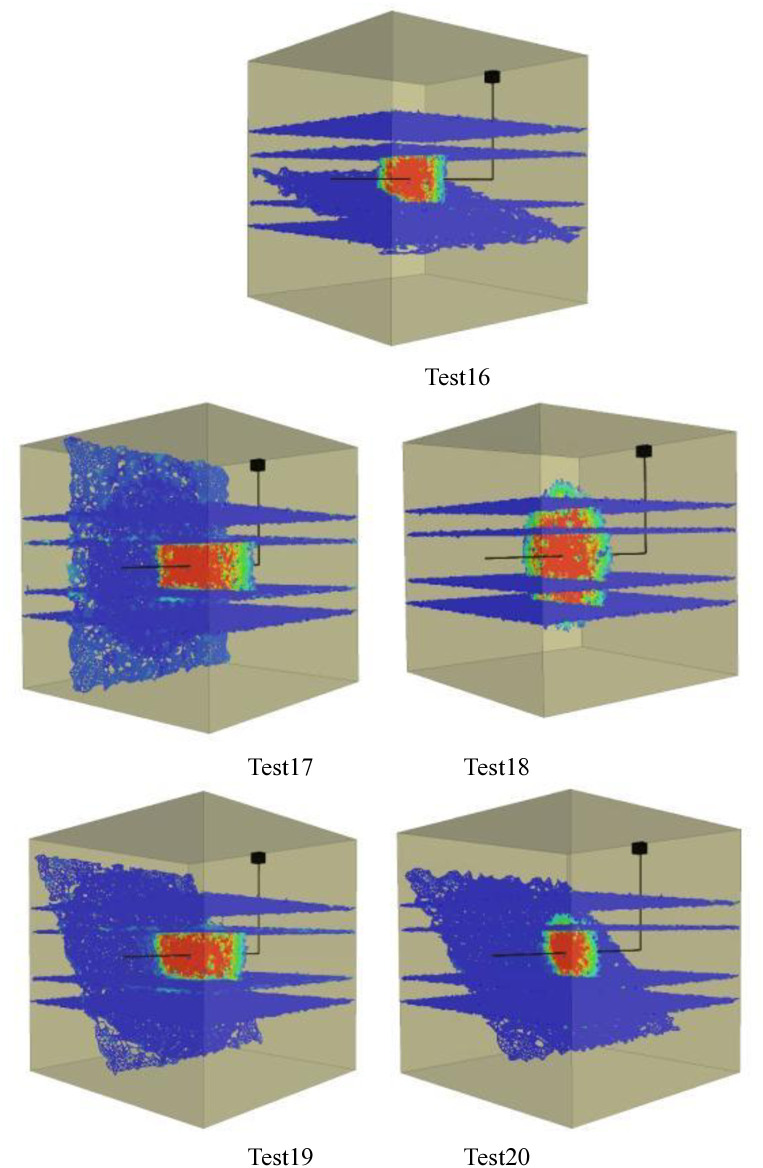
Graph of orthogonal test results(Bedding number 4). Test16, Test17, Test18, Test19, Test20.

**Fig 5:**
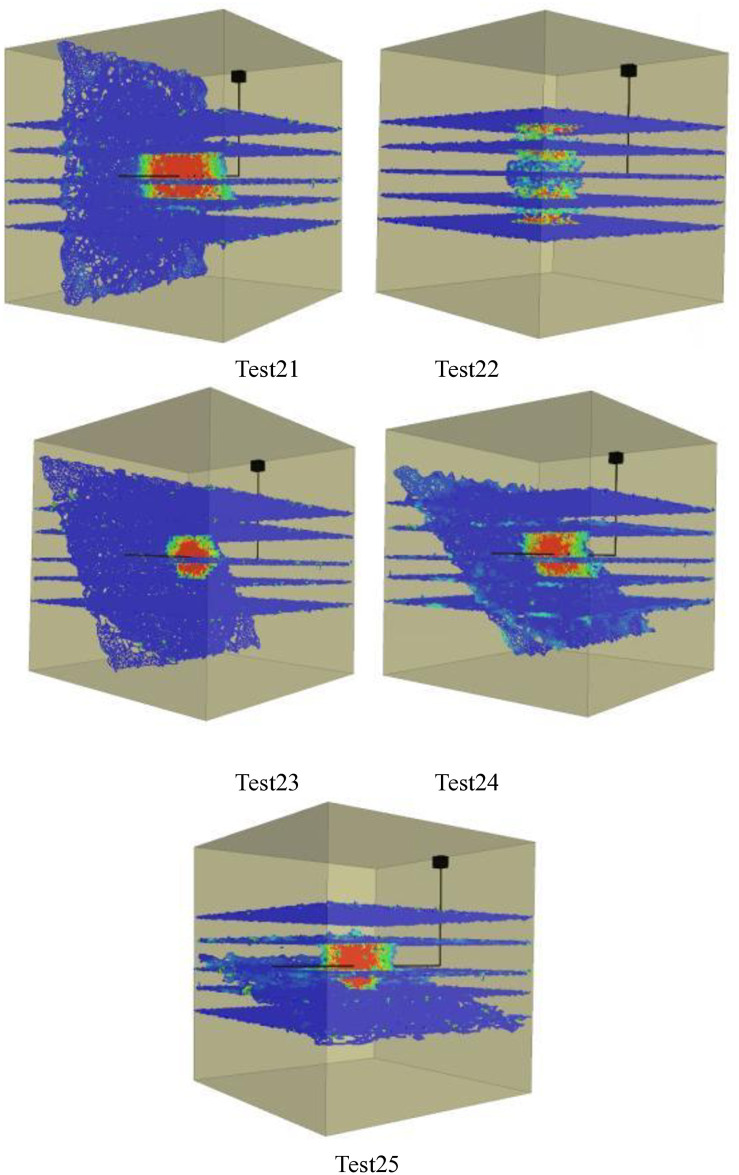
Graph of orthogonal test results(Bedding number 5). Test21, Test22, Test23, Test24, Test25.

Figs 1–5 shows the orthogonal test results. Blue in the figure is the natural weak surface (bedding or high Angle fractures), while the cloud image shows the expansion of hydraulic fractures, which may pass through the bedding or high Angle fractures, or may be captured. Through the Xsite simulation software, the fracture characteristics can be obtained, such as the total fracture area, fracture volume, shear fracture area and tensile fracture area. According to the fracture characteristics and the principle of orthogonal test, the main controlling factors affecting the fracture propagation of hydraulic fracturing can be obtained, and the optimal fracturing scheme can be obtained.

### 5.2 Based on the volume of the crack morphology

The fracture volume is the direct result of reservoir reconstruction. The purpose of hydraulic fracturing is to form fractures in the reservoir by injecting high-pressure liquid. The fracture volume is the new pore space formed by fracturing, which directly reflects the effect of reservoir reconstruction. The larger the fracture volume, the more fully fractured the reservoir, the more fluid flow paths through the fracture, and the higher the oil and gas productivity. According to the test results, the crack morphology and volume characteristic parameters under different test conditions were obtained, as shown in Table 4.

**Table 4:** Characteristic parameters of crack morphology and volume under different test conditions.

Level Factor	Bedding number	Bedding friction Angle/°	High angle fracture dip angle/°	Injection rate/m^3^/min	Viscosity/mPa.s	σv-σh/MPa	Volume of the crack morphology/m^3^
Test1	1	1	0	2	5	5	4.98
Test2	1	15	30	3	10	7.5	11.1
Test3	1	30	45	4	15	10	12.4
Test4	1	45	75	5	20	12.5	12.5
Test5	1	60	90	6	25	15	12.7
Test6	2	1	30	4	20	15	8.42
Test7	2	15	45	5	25	5	10
Test8	2	30	75	6	5	7.5	16.1
Test9	2	45	90	2	10	10	6.97
Test10	2	60	0	3	15	12.5	8.2
Test11	3	1	45	6	10	12.5	17
Test12	3	15	75	2	15	15	7.62
Test13	3	30	90	3	20	5	10.9
Test14	3	45	0	4	25	7.5	13.4
Test15	3	60	30	5	5	10	14
Test16	4	1	75	3	25	10	7.63
Test17	4	15	90	4	5	12.5	12.9
Test18	4	30	0	5	10	15	12.9
Test19	4	45	30	6	15	5	13.2
Test20	4	60	45	2	20	10	6.86
Test21	5	1	90	5	15	7.5	15.1
Test22	5	15	0	6	20	10	20.4
Test23	5	30	30	2	25	12.5	20.4
Test24	5	45	45	3	5	15	11.8
Test25	5	60	75	4	10	5	15.3
Mean value1	10.736	10.626	11.976	9.366	11.956	10.876	
Mean value2	9.938	12.404	13.424	9.926	12.654	13.925	
Mean value3	12.584	14.54	11.612	12.484	11.304	11.377	
Mean value4	10.698	11.574	11.83	12.9	11.816	14.2	
Mean value5	16.6	11.412	11.714	15.88	12.826	10.688	
Standard deviation1	2.93	4.61	5.24	5.59	3.77	3.48	
Standard deviation2	3.23	4.40	3.99	2.25	3.48	1.90	
Standard deviation3	3.15	3.39	3.32	2.26	2.91	4.89	
Standard deviation4	2.83	2.37	3.64	1.71	4.71	4.17	
Standard deviation5	3.34	3.30	2.72	2.79	4.31	2.22	
Range	0.51	2.24	2.51	3.88	1.80	2.99	

As can be seen from Table 4, the ranges corresponding to the six influencing factors in descending order are fracturing fluid injection rate, vertical stress difference, high-angle crack inclination, internal friction Angle of bedding, fracturing fluid viscosity, and bedding quantity. It shows that the influence degree of the six influencing factors on the hydraulic crack morphology volume is in order of fracturing fluid injection rate > vertical stress difference > high Angle crack inclination > internal friction Angle of bedding > fracturing fluid viscosity > bedding quantity.

**Fig 6:**
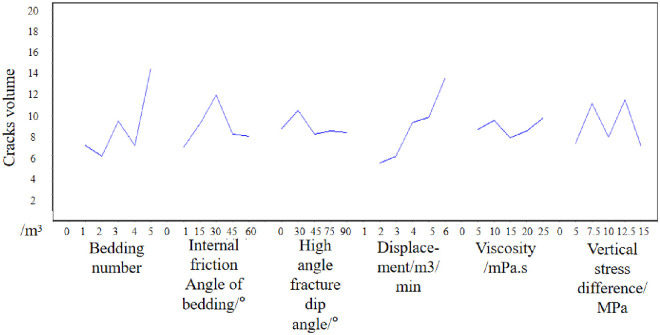
Effect diagram(Based on the volume of the crack morphology).

Then the mean of each factor is analyzed. In the number of bedding, the mean value of 5 is the largest, which indicates that when the number of bedding is 5, the growth of hydraulic crack shape volume is the most favorable. Then, when the bedding friction Angle is 30°, the high Angle crack inclination is 30°, the injection rate is 6m3/min, the viscosity is 25mPa·s, and the vertical stress difference is 7.5MPa, the growth of the hydraulic crack morphology volume is the most favorable. The effect curve is shown in Fig 6. Fig 6 is the effect diagram drawn according to the orthogonal test, reflecting the influence trend of different influencing factors on the crack evaluation index.

Therefore, from the perspective of crack morphology and volume, the optimal crack design scheme is as follows: the layer rational number is 5, the bedding friction Angle is 1°, the high Angle crack inclination is 0°, the injection rate is 2m3/min, the viscosity is 20mPa·s, and the vertical stress difference is 10MPa.

### 5.3 Based on the total area of cracks

After hydraulic fracturing, the total fracture area directly affects the permeability of the reservoir. The more fractures and the larger the total area, the more fluid flow paths in the reservoir, the higher the permeability, thus increasing the oil and gas production. The total fracture area is an important parameter for evaluating reservoir conductivity. The large fracture area means that there are more diversion channels, which can more effectively transport oil and gas from the reservoir to the wellbore. The complexity of fracture network can be preliminarily assessed by the total fracture area. A larger fracture area usually means a more complex fracture network, which can cover the reservoir more extensively, adding more contact surfaces, and thus improving recovery. The increase of fracture area means the increase of fracture length, width and number. The larger fracture area provides more paths for oil and gas flow, especially in low permeability reservoirs. According to the test results, the characteristic parameters of the total crack area under different test conditions were obtained, as shown in Table 5.

**Table 5:** The characteristic parameters of the total crack area under different test conditions.

Level Factor	Bedding number	Bedding friction Angle/°	High angle fracture dip angle/°	Injection rate/m^3^/min	Viscosity/mPa.s	σv-σh/MPa	Total area of cracks/m^2^
Test1	1	1	0	2	5	5	394.49
Test2	1	15	30	3	10	7.5	1730
Test3	1	30	45	4	15	10	1800
Test4	1	45	75	5	20	12.5	1360
Test5	1	60	90	6	25	15	1500
Test6	2	1	30	4	20	15	2000
Test7	2	15	45	5	25	5	2180
Test8	2	30	75	6	5	7.5	2420
Test9	2	45	90	2	10	10	1160
Test10	2	60	0	3	15	12.5	552
Test11	3	1	45	6	10	12.5	2770
Test12	3	15	75	2	15	15	1020
Test13	3	30	90	3	20	5	1190
Test14	3	45	0	4	25	7.5	1080
Test15	3	60	30	5	5	10	4850
Test16	4	1	75	3	25	10	1600
Test17	4	15	90	4	5	12.5	2330
Test18	4	30	0	5	10	15	840
Test19	4	45	30	6	15	5	2260
Test20	4	60	45	2	20	10	1180
Test21	5	1	90	5	15	7.5	2070
Test22	5	15	0	6	20	10	1830
Test23	5	30	30	2	25	12.5	1830
Test24	5	45	45	3	5	15	2440
Test25	5	60	75	4	10	5	4050
Mean value1	1356.898	1766.898	939.298	1116.898	2486.898	2014.898	
Mean value2	1662.4	1818	2534	1502.4	2110	1825	
Mean value3	2182	1616	2074	2252	1540.4	2070	
Mean value4	1642	1660	2090	2260	1512	1768.4	
Mean value5	2444	2426.4	1650	2156	1638	1560	
Standard deviation1	506.41	782.79	503.95	457.30	1413.77	1227.61	
Standard deviation2	698.75	1412.00	1171.85	635.94	1171.24	494.49	
Standard deviation3	1484.09	549.49	622.68	987.96	650.15	1271.48	
Standard deviation4	585.44	573.55	1083.37	1383.98	339.49	770.56	
Standard deviation5	833.44	1699.12	471.81	445.90	364.00	596.93	
Range	977.68	1149.62	700.04	938.08	1074.28	776.99	

As can be seen from Table 5, the ranges corresponding to the six influencing factors are, in descending order, internal friction Angle of bedding, fracturing fluid viscosity, bedding quantity, fracturing fluid injection rate, vertical stress difference, and high-angle crack inclination Angle. It shows that the influence degree of the six influencing factors on the total area of hydraulic cracks is in the order of internal friction Angle of bedding > fracturing fluid viscosity > bedding quantity > fracturing fluid injection rate > vertical stress difference > high Angle crack inclination.

**Fig 7:**
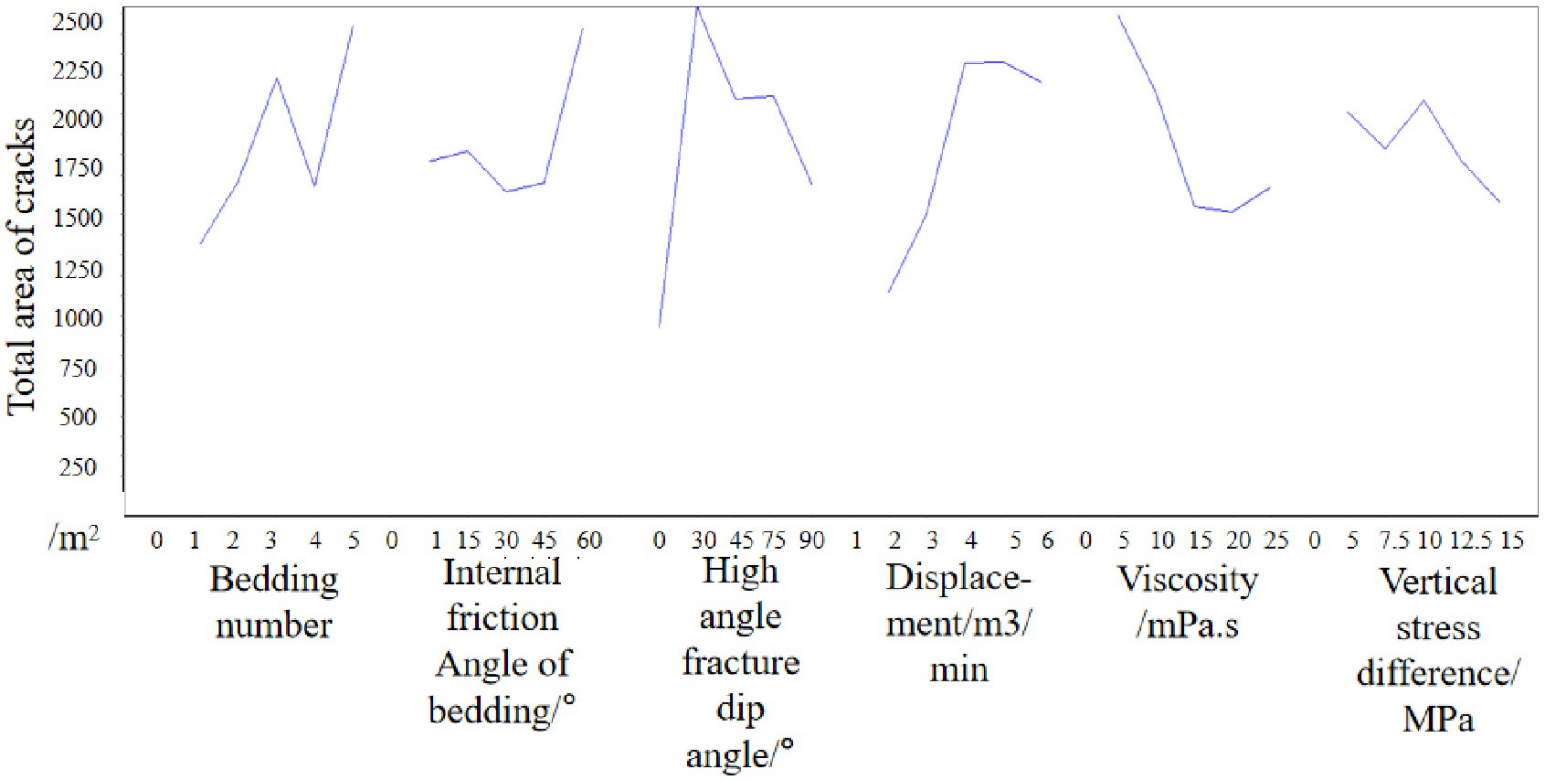
Effect diagram(Based on the total area of cracks).

Then the mean of each factor is analyzed. In the number of bedding, the mean value of 3 is the largest, which indicates that when the number of bedding is 3, the total area of hydraulic cracks is the most favorable to increase. Then, when the bedding friction Angle is 60°, the high Angle crack inclination is 30°, the injection rate is 5m3/min, the viscosity is 5mPa·s, and the vertical stress difference is 5MPa, the total area of hydraulic crack is most conducive to the growth of hydraulic crack. The effect curve is shown in Fig 7.

Therefore, from the perspective of the total crack area, the optimal crack design scheme is as follows: the number of layers is 3, the bedding friction Angle is 60°, the high-angle crack inclination is 30°, the injection rate is 5m3/min, the viscosity is 5mPa·s, and the vertical stress difference is 10MPa.

### 5.4 Based on tensile cracks area

In the process of hydraulic fracturing, cracks will open under the action of high pressure fluid, resulting in tensile fractures. Therefore, tensile fractures occupy a large proportion in hydraulic fractures, and are also an important basis for measuring the effect of reservoir reconstruction. According to the test results, the characteristic parameters of tensile crack area under different test conditions were obtained, as shown in Table 6.

**Table 6:** Characteristic parameters of tensile crack area under different test conditions.

Level Factor	Bedding number	Bedding friction Angle/°	High angle fracture dip angle/°	Injection rate/m^3^/min	Viscosity/mPa.s	σv-σh/MPa	Tensile crack area/m^2^
Test1	1	1	0	2	5	5	310.69
Test2	1	15	30	3	10	7.5	250
Test3	1	30	45	4	15	10	282
Test4	1	45	75	5	20	12.5	346
Test5	1	60	90	6	25	15	410
Test6	2	1	30	4	20	15	301
Test7	2	15	45	5	25	5	231
Test8	2	30	75	6	5	7.5	322
Test9	2	45	90	2	10	10	123
Test10	2	60	0	3	15	12.5	435
Test11	3	1	45	6	10	12.5	315
Test12	3	15	75	2	15	15	111
Test13	3	30	90	3	20	5	136
Test14	3	45	0	4	25	7.5	796
Test15	3	60	30	5	5	10	222
Test16	4	1	75	3	25	10	207
Test17	4	15	90	4	5	12.5	257
Test18	4	30	0	5	10	15	673
Test19	4	45	30	6	15	5	282
Test20	4	60	45	2	20	10	146
Test21	5	1	90	5	15	7.5	186
Test22	5	15	0	6	20	10	1290
Test23	5	30	30	2	25	12.5	1290
Test24	5	45	45	3	5	15	2290
Test25	5	60	75	4	10	5	205
Mean value1	319.738	263.938	700.938	396.138	680.338	232.938	
Mean value2	282.4	427.8	469	663.6	313.2	388.5	
Mean value3	316	540.6	652.8	368.2	259.2	378.333	
Mean value4	313	767.4	238.2	331.6	443.8	528.6	
Mean value5	1052.2	283.6	222.4	523.8	586.8	757	
Standard deviation1	55.15	55.65	340.54	452.72	805.65	61.05	
Standard deviation2	103.20	437.37	411.39	786.70	190.44	240.14	
Standard deviation3	250.46	414.12	815.28	216.31	108.87	410.97	
Standard deviation4	185.92	793.42	85.91	178.94	431.15	385.03	
Standard deviation5	789.09	116.45	104.94	385.44	409.96	787.76	
Range	733.94	737.77	729.38	607.77	696.78	726.71	

As can be seen from Table 6, the ranges corresponding to the six influencing factors are, in descending order, internal friction Angle of bedding, number of bedding, high Angle crack inclination, vertical stress difference, fracturing fluid viscosity, and fracturing fluid injection rate. It shows that the influence degree of the six influencing factors on the tensile crack area of hydraulic cracks is in the order of internal friction Angle > bedding quantity > high Angle crack inclination > vertical stress difference > fracturing fluid viscosity > fracturing fluid injection rate.

**Fig 8:**
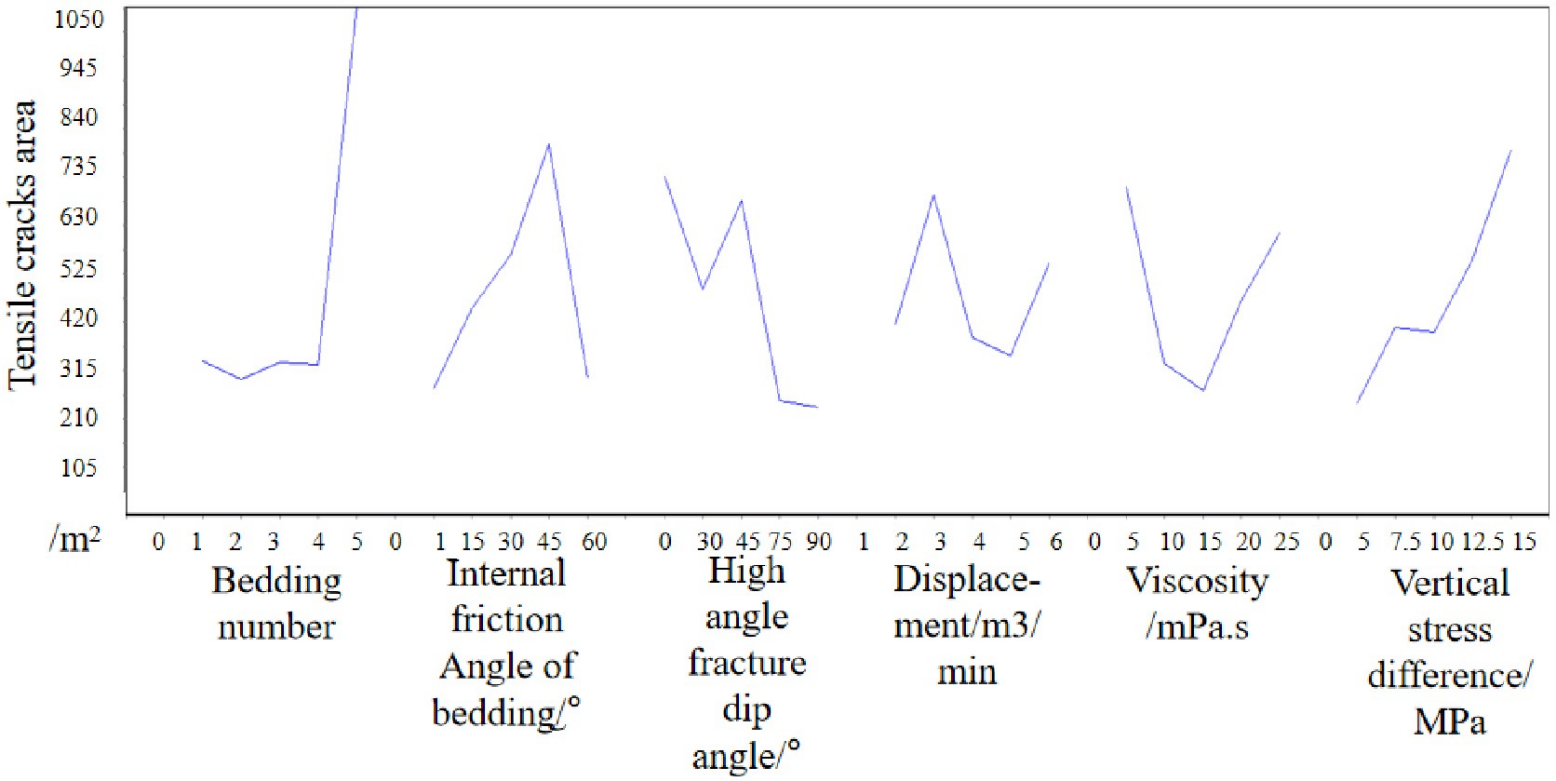
Effect diagram(Based on tensile cracks area).

Then the mean of each factor is analyzed. In the number of bedding, the mean value of 5 is the largest, which indicates that when the number of bedding is 5, it is the most beneficial to the growth of the tensile crack area. Then, when the bedding friction Angle is 45°, the high Angle crack inclination is 0°, the injection rate is 3m3/min, the viscosity is 5mPa·s, and the vertical stress difference is 15MPa, the tensile crack area of the hydraulic crack is the most favorable. The effect curve is shown in Fig 8.

Therefore, from the perspective of tensile crack area, the optimal crack design scheme is as follows: the number of layers is 5, the bedding friction Angle is 45°, the high Angle crack inclination is 30°, the injection rate is 3m3/min, the viscosity is 5mPa·s, and the vertical stress difference is 15MPa.

### 5.5 Based on shear crack area

As mentioned above, tensile fractures account for an important proportion in hydraulic fractures. However, due to the uneven distribution of in-situ stress, tensile fractures will appear in the uneven in-situ stress at the same time. Therefore, shear fractures in the process of hydraulic fracturing cannot be ignored. According to the test results, the characteristic parameters of shear crack area under different test conditions were obtained, as shown in Table 7.

**Table 7:** Characteristic parameters of shear crack area under different test conditions.

Level Factor	Bedding number	Bedding friction Angle/°	High angle fracture dip angle/°	Injection rate/m^3^/min	Viscosity/mPa.s	σv-σh/MPa	Shear crack area/m^2^
Test1	1	1	0	2	5	5	83.8
Test2	1	15	30	3	10	7.5	1480
Test3	1	30	45	4	15	10	1510
Test4	1	45	75	5	20	12.5	1010
Test5	1	60	90	6	25	15	1090
Test6	2	1	30	4	20	15	1700
Test7	2	15	45	5	25	5	1950
Test8	2	30	75	6	5	7.5	2100
Test9	2	45	90	2	10	10	1040
Test10	2	60	0	3	15	12.5	117
Test11	3	1	45	6	10	12.5	2450
Test12	3	15	75	2	15	15	904
Test13	3	30	90	3	20	5	1060
Test14	3	45	0	4	25	7.5	279
Test15	3	60	30	5	5	10	4630
Test16	4	1	75	3	25	10	1400
Test17	4	15	90	4	5	12.5	2080
Test18	4	30	0	5	10	15	168
Test19	4	45	30	6	15	5	1980
Test20	4	60	45	2	20	10	1030
Test21	5	1	90	5	15	7.5	1880
Test22	5	15	0	6	20	10	540
Test23	5	30	30	2	25	12.5	540
Test24	5	45	45	3	5	15	2290
Test25	5	60	75	4	10	5	3850
Mean value1	1034.76	1502.76	237.56	719.56	2236.76	1784.76	
Mean value2	1381.4	1390.8	2066	1269.4	1797.6	1434.75	
Mean value3	1864.6	1075.6	1846	1883.8	1278.2	1691.667	
Mean value4	1331.6	1319.8	1852.8	1927.6	1068	1239.4	
Mean value5	1820	2143.4	1430	1632	1051.8	1230.4	
Standard deviation1	516.16	787.72	165.05	365.92	1442.84	1244.92	
Standard deviation2	728.90	1534.53	1370.22	826.07	1262.41	703.32	
Standard deviation3	1553.99	685.41	622.50	1153.54	692.36	1350.35	
Standard deviation4	697.16	725.90	1083.10	1499.59	369.62	891.48	
Standard deviation5	1234.68	1763.59	453.78	706.47	598.10	720.86	
Range	1037.83	1078.18	1205.17	793.13	1073.22	647.04	

As can be seen from Table 7, the ranges corresponding to the six influencing factors are, from the largest to the smallest, the internal friction Angle of bedding, the number of bedding, the viscosity of fracturing fluid, the Angle of high crack Angle, the injection rate of fracturing fluid, and the vertical stress difference. The results show that the influence degree of the six influencing factors on the shear area of hydraulic cracks is in the order of high Angle crack inclination > internal friction Angle of bedding > fracturing fluid viscosity > number of bedding > fracturing fluid injection rate > vertical stress difference.

**Fig 9:**
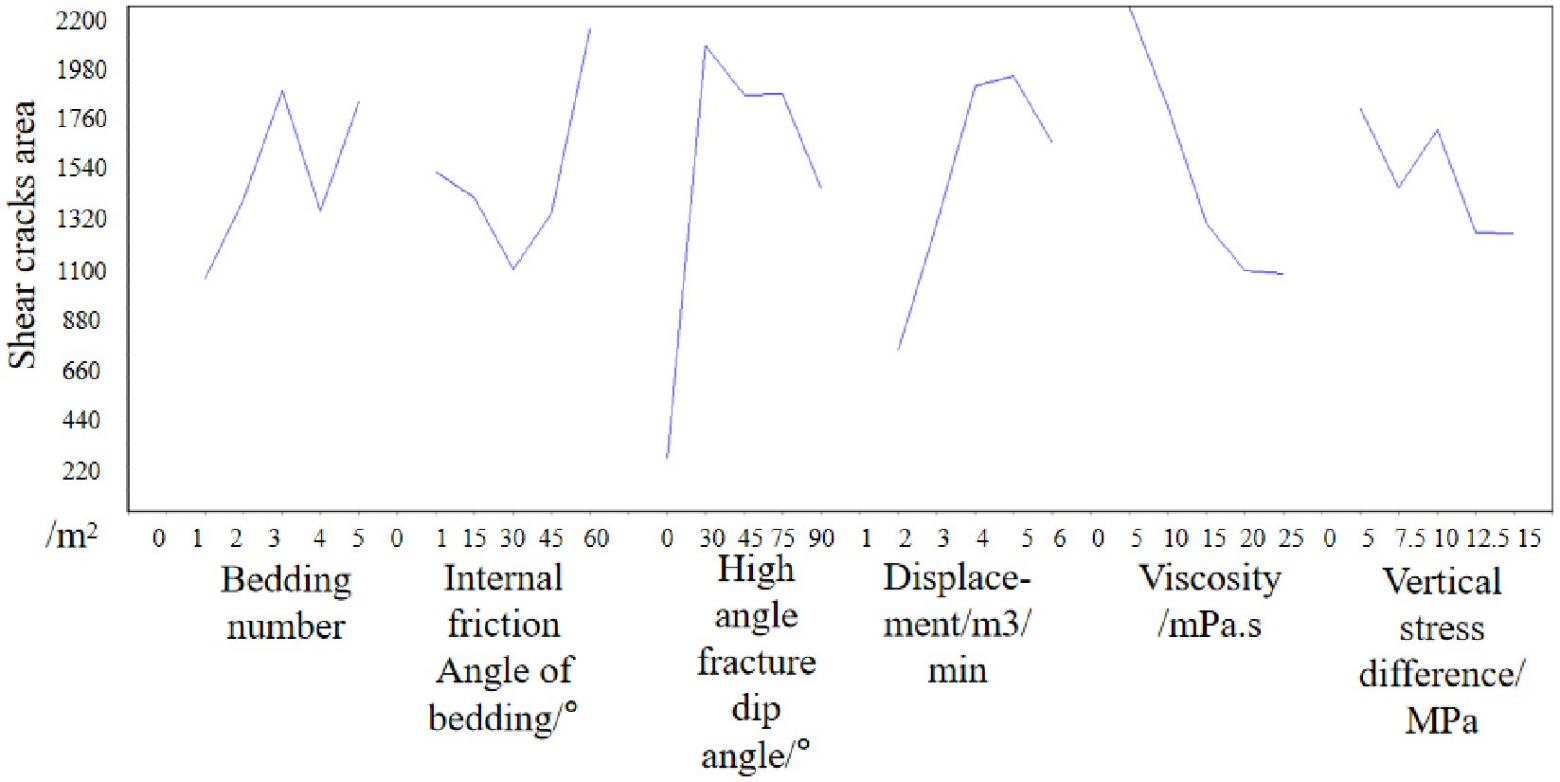
Effect diagram(Based on shear crack area).

Then the mean of each factor is analyzed. In the number of bedding, the mean value of 3 is the largest, which indicates that when the number of bedding is 5, the shear area of hydraulic crack is the most favorable. Then, when the bedding friction Angle is 60°, the high Angle crack inclination is 30°, the injection rate is 5m^3^/min, the viscosity is 5mPa·s, and the vertical stress difference is 5MPa, the shear area of the hydraulic crack is the most favorable. The effect curve is shown in Fig 9. The shear fracture area increased sharply in the range of 0–30°, this is because when the Angle of the high-angle crack changes from 0° to 30°, the high-angle crack grows from nothing, and the emergence of the high-angle crack can interact with the hydraulic crack, resulting in a large number of shear cracks and natural cracks.

Therefore, from the perspective of crack shear area, the optimal crack design scheme is as follows: the bedding number is 3, the bedding friction Angle is 60°, the high-angle crack inclination is 30°, the injection rate is 5m^3^/min, the viscosity is 5mPa·s, and the vertical stress difference is 10MPa.

### 5.6 Optimal fracturing scheme design considering multiple quantitative indexes

The weight analysis method was used to comprehensively consider the influence of four quantitative indexes on the main controlling factors of complex crack formation. The integration test results are shown in Table 8.

**Table 8:** Integration of test results.

Quantitative index	Influence factor	Range	Weight
Based on the volume of the crack morphology	Bedding number	0.51m^3^	3.66%
	Bedding friction Angle	2.24m^3^	16.08%
	High angle fracture dip angle	2.51m^3^	18.02%
	Injection rate	3.88m^3^	27.85%
	Viscosity	1.8m^3^	12.92%
	σ_v_-σ_h_	2.99m^3^	21.46%
Based on the total area of cracks	Bedding number	977.68m^2^	17.41%
	Bedding friction Angle	1149.62m^2^	20.47%
	High angle fracture dip angle	700.04m^2^	12.46%
	Injection rate	938.08m^2^	16.70%
	Viscosity	1074.28m^2^	19.13%
	σ_v_-σ_h_	776.99m^2^	13.83%
Based on tensile cracks area	Bedding number	733.94m^2^	17.34%
	Bedding friction Angle	737.77m^2^	17.43%
	High angle fracture dip angle	729.38m^2^	17.23%
	Injection rate	607.77m^2^	14.36%
	Viscosity	696.78m^2^	16.46%
	σ_v_-σ_h_	726.71m^2^	17.17%
Based on shear cracks area	Bedding number	1037.83m^2^	17.79%
	Bedding friction Angle	1078.18m^2^	18.48%
	High angle fracture dip angle	1205.17m^2^	20.66%
	Injection rate	793.13m^2^	13.59%
	Viscosity	1073.22m^2^	18.39%
	σ_v_-σ_h_	647.04m^2^	11.09%

Table 9 shows the optimal fracturing plan design considering the weight of various factors

**Table 9:** The optimal fracturing scheme design considering various factors influencing weight.

H F	Quantitative basis	Standard deviation1/weight	Standard deviation2/weight	Standard deviation3/weight	Standard deviation4/weight	Standard deviation5/weight	Optimal solution
Bedding number	Volume of the crack morphology	2.93/ 18.93%	3.23/ 20.87%	3.15/ 20.35%	2.83/ 18.28%	3.34/ 21.58%	5
	Total area of cracks	506.41/ 12.33%	698.75/ 17.01%	1484.09/ 36.13%	585.44/ 14.25%	833.44/ 20.29%	
	Tensile cracks area	55.15/ 3.99%	103.20/ 7.46%	250.46/ 18.10%	185.92/ 13.44%	789.09/ 57.02%	
	Shear cracks area	516.16/ 10.91%	728.90/ 15.41%	1553.99/ 32.85%	697.16/ 14.74%	1234.68/ 26.10%	
	Total impact weight	11.54%	15.18%	26.86%	15.18%	31.25%	
Bedding friction Angle	Volume of the crack morphology	4.61/ 25.51%	4.40/ 24.35%	3.39/ 18.76%	2.37/ 13.12%	3.30/ 18.26%	15°
	Total area of cracks	782.79/ 15.60%	1412.00/ 28.14%	549.49/ 10.95%	573.55/ 11.43%	1699.12/ 33.87%	
	Tensile cracks area	55.65/ 3.06%	437.37/ 24.07%	414.12/ 22.79%	793.42/ 43.67%	116.45/ 6.41%	
	Shear cracks area	787.72/ 14.33%	1534.53/ 27.92%	685.41/ 12.47%	725.90/ 13.21%	1763.59 32.08%	
	Total impact weight	14.63%	26.12%	16.24%	20.35%	22.66%	
High angle fracture dip angle	Volume of the crack morphology	5.24/ 27.71%	3.99/ 21.10%	3.32/ 17.56%	3.64/ 19.25%	2.72/ 14.38%	30°
	Total area of cracks	503.95/ 13.08%	1171.85/ 30.41%	622.68/ 16.16%	1083.37/ 28.11%	471.81/ 12.24%	
	Tensile cracks area	340.54/ 19.37%	411.39/ 23.40%	815.28/ 46.37%	85.91/ 4.89%	104.94/ 5.97%	
	Shear cracks area	165.05/ 4.47%	1370.22/ 37.09%	622.50/ 16.85%	1083.10/ 29.32%	453.78/ 12.28%	
	Total impact weight	16.16%	28.00%	24.23%	20.39%	11.22%	
Injection rate	Volume of the crack morphology	5.59/ 38.29%	2.25/ 15.41%	2.26/ 15.48%	1.71/ 11.71%	2.79/ 19.11%	5m^3^/min
	Total area of cracks	457.30/ 11.69%	635.94/ 16.26%	987.96/ 25.26%	1383.98/ 35.39%	445.90/ 11.40%	
	Tensile cracks area	452.72/ 22.41%	786.70/ 38.94%	216.31/ 10.71%	178.94/ 8.86%	385.44/ 19.08%	
	Shear cracks area	365.92/ 8.04%	826.07/ 18.15%	1153.54/ 25.34%	1499.59/ 32.95%	706.47/ 15.52%	
	Total impact weight	20.11%	22.19%	19.20%	22.23%	16.28%	
Viscosity	Volume of the crack morphology	3.77/ 19.66%	3.48/ 18.14%	2.91/ 15.17%	4.71/ 24.56%	4.31/ 22.47%	5mPa.s
	Total area of cracks	1413.77/ 35.89%	1171.24/ 29.74%	650.15/ 16.51%	339.49/ 8.62%	364.00/ 9.24%	
	Tensile cracks area	805.65/ 41.40%	190.44/ 9.79%	108.87/ 5.59%	431.15/ 22.15%	409.96/ 21.07%	
	Shear cracks area	1442.84/ 33.05%	1262.41/ 28.92%	692.36/ 15.86%	369.62/ 8.47%	598.10/ 13.70%	
	Total impact weight	32.50%	21.65%	13.28%	15.95%	16.62%	
σ_v_-σ_h_	Volume of the crack morphology	3.48/ 20.89%	1.90/ 11.40%	4.89/ 29.35%	4.17/ 25.03%	2.22/ 13.33%	10MPa
	Total area of cracks	1227.61/ 28.15%	494.49/ 11.34%	1271.48/ 29.16%	770.56/ 17.67%	596.93/ 13.69%	
	Tensile cracks area	61.05/ 3.24%	240.14/ 12.74%	410.97/ 21.80%	385.03/ 20.43%	787.76/ 41.79%	
	Shear cracks area	1244.92/ 25.35%	703.32/ 14.32%	1350.35/ 27.50%	891.48/ 18.15%	720.86/ 14.68%	
	Total impact weight	19.41%	12.45%	26.95%	20.32%	20.87%	

So as can been seen from the Table 9, Considering the weight of various quantization criteria, the optimal fracturing scheme has 5 layer rationals, 1° bedding friction Angle, 0° high-angle crack inclination, 2m^3^/min injection rate, 20mPa·s viscosity, and 10MPa vertical stress difference. b. From the perspective of the total crack area, the optimal crack design scheme is as follows: the number of layers is 5, the bedding friction Angle is 30°, the high-angle crack inclination is 30°, the injection rate is 5m^3^/min, the viscosity is 5mPa·s, and the vertical stress difference is 10MPa.

### 5.7 Combined engineering verification

#### 5.7.1 Case well basic information

**Fig 10:**
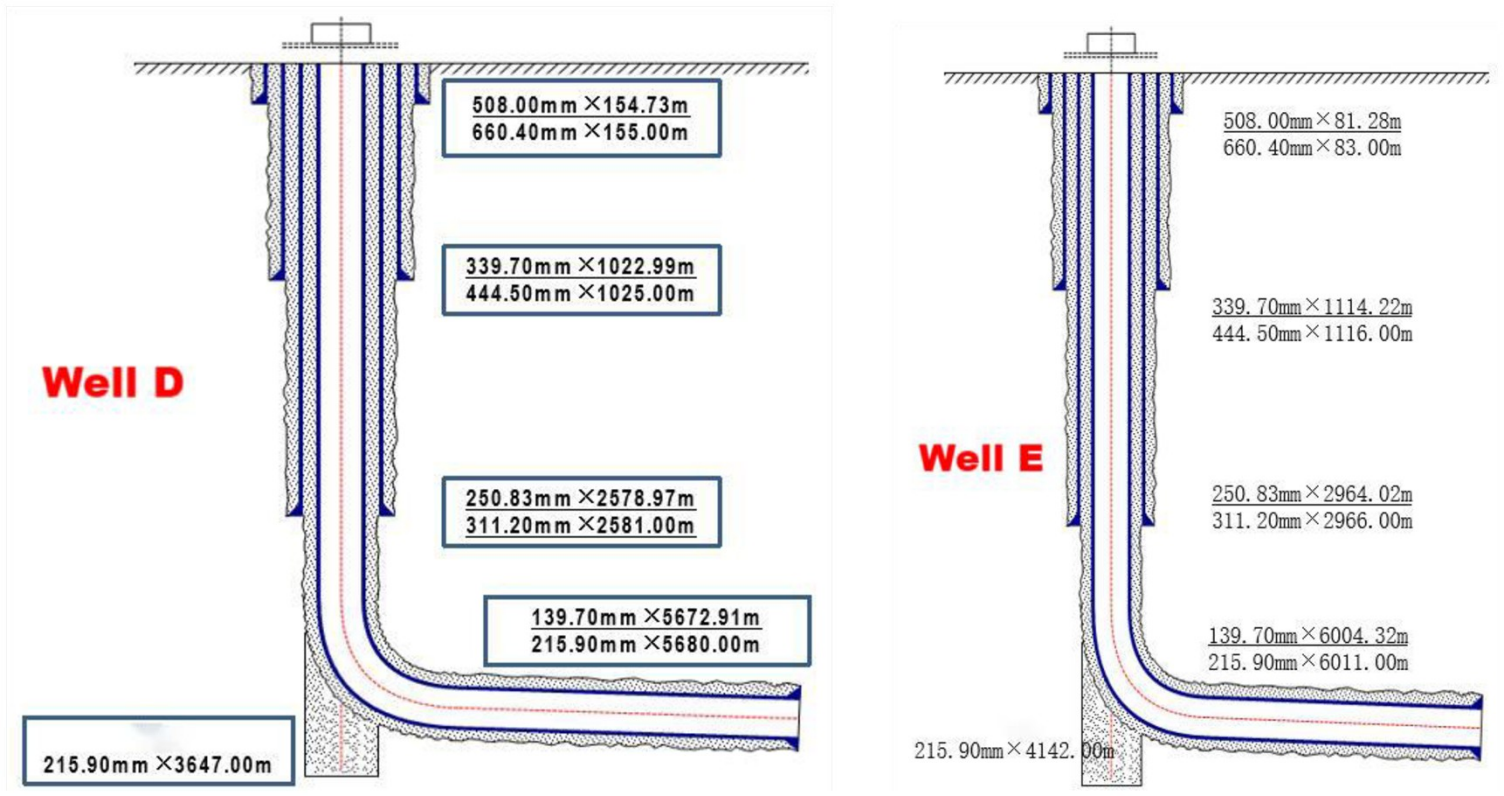
Real drilling depth structure diagram. (a) Well D, (b) Well E.

Well D is located in Group 6, Gufoshan Village, Qingsheng Town, Rongchang District, Chongqing, with an artificial bottom of 5621m; Well E is located in Group 9, Quanyi Village, Yunding Town, Longchang City, Neijiang City, Sichuan Province, with an artificial well bottom of 5942m. As shown in Fig 10.

The core of well D has been taken for 7 times from Longmaxi Formation to Baata Formation, with a core penetration of 122.5m, a core length of 122.5m, and a yield of 100%. The core observation and statistics of this well show that there are 1,108 cracks in the core section of 3478.0–3600.5m, mainly filled horizontal fractures, and fractures in the middle and upper part of the 4 small formations and in the 2 small formations—Wufeng Formation.

In well E, 12 barrels of coring were taken from Longmaxi Formation and Baata Formation, the coring footage was 189.94m, the core length was 189.65m, and the harvest rate was 99.85%. The core observation statistics showed that 1007 cracks were developed in the coring well section of 3905.0–4096.2m, mainly unfilled vertical and inclined cracks. Cracks developed in the four small layers of faults, and the seam density was 30.4 /m. 1–2 small layer cracks developed with a seam density of 2.9 cracks /m.

As indicated by Stoneley wave attenuation in well D, there are four possible micro-cracks in the horizontal section with a cumulative length of 462m. The comprehensive fracture interpretation of well E logging indicates that there are four possible micro-fractures in the horizontal section, with a cumulative length of 58m.

#### 5.7.2 Fracturing operation

30 stages of well D have been designed, and 30 stages have been fractured. The construction displacement is 16~18m³/min, the construction pressure is 75~112MPa, the average liquid strength is 29.43m³/m, and the sand strength is 2.00t/m, which meets the design requirements. Table 10 describes the fracturing parameters.

**Table 10:** Fracturing parameter design of D well.

	Design parameters	Actual parameters
Fracturing stage length	1800m	1800m
Number of stages	30(60.0m)	30(60.0m)
Fracturing fluid rate	14~18m^3^/min	14~18m^3^/min
Proppant combination	60%Quartz sand+40%Ceramite	59.3%Quartz sand+40.7%Ceramite
Sanding	Body segment2.0t/m, Risk segment2.0t/m	Body segment2.00t/m, Risk segment2.00

**Fig 11:**
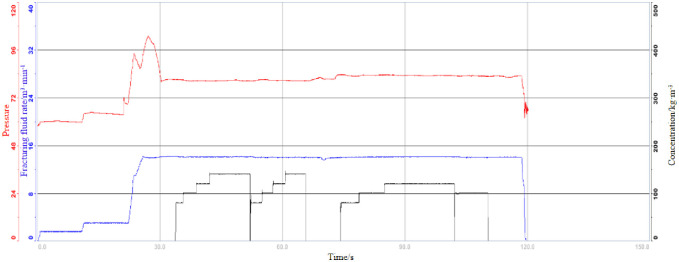
Fracturing construction drawing of section 19 of D well.

Fig 11 Construction curve analysis: During the fracturing process, the construction pressure is stable, the filtration loss is not obvious, the sand is added smoothly, and there is no natural crack feature. Fracturing monitoring analysis: During stage 19 fracturing, a strong energy event response was detected near the east side, indicating natural fracture development.

The extended pressure gradient is estimated to be 0.027MPa/m according to the stop pumping pressure of adjacent Wells, and the fracturing fluid is predicted according to the resistance reduction rate of 70%. When the displacement is about 18m3/min, the predicted construction pressure is 103.93~116.76MPa.

**Fig 12:**
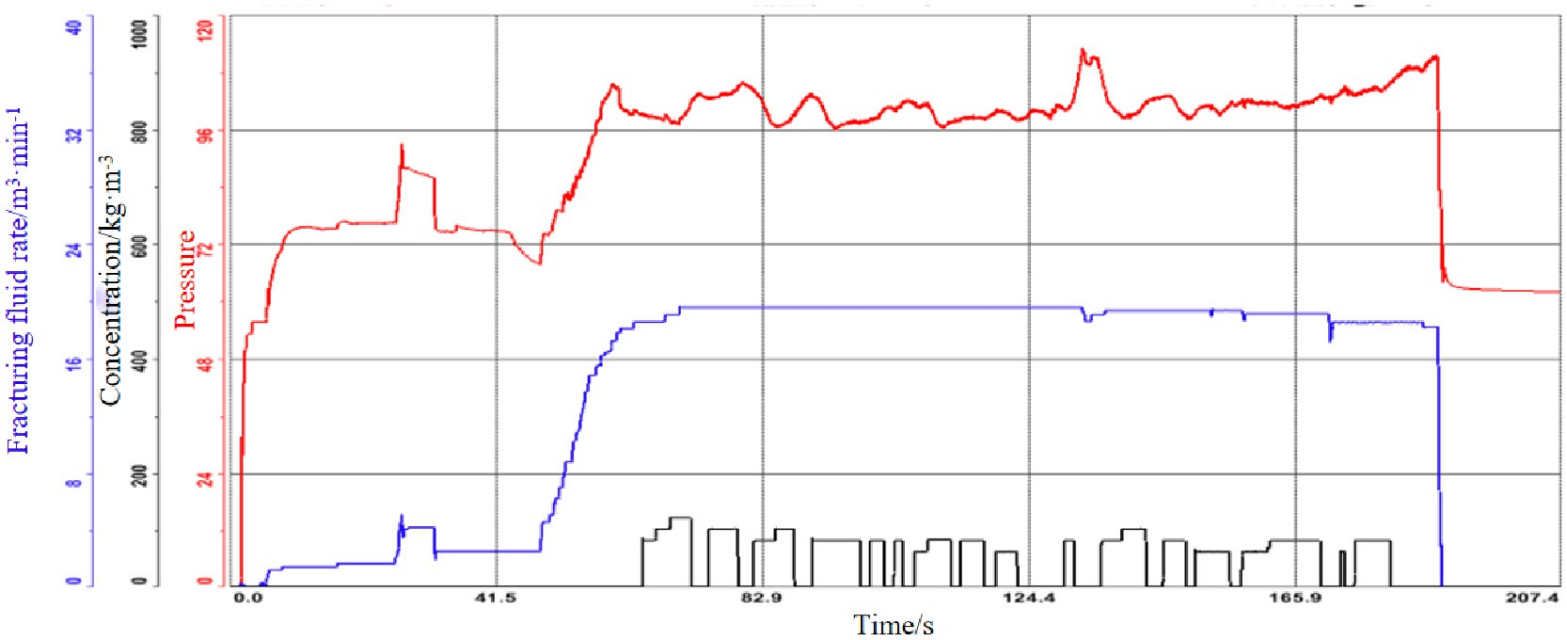
Sand strength 2.3t/m.

**Fig 13:**
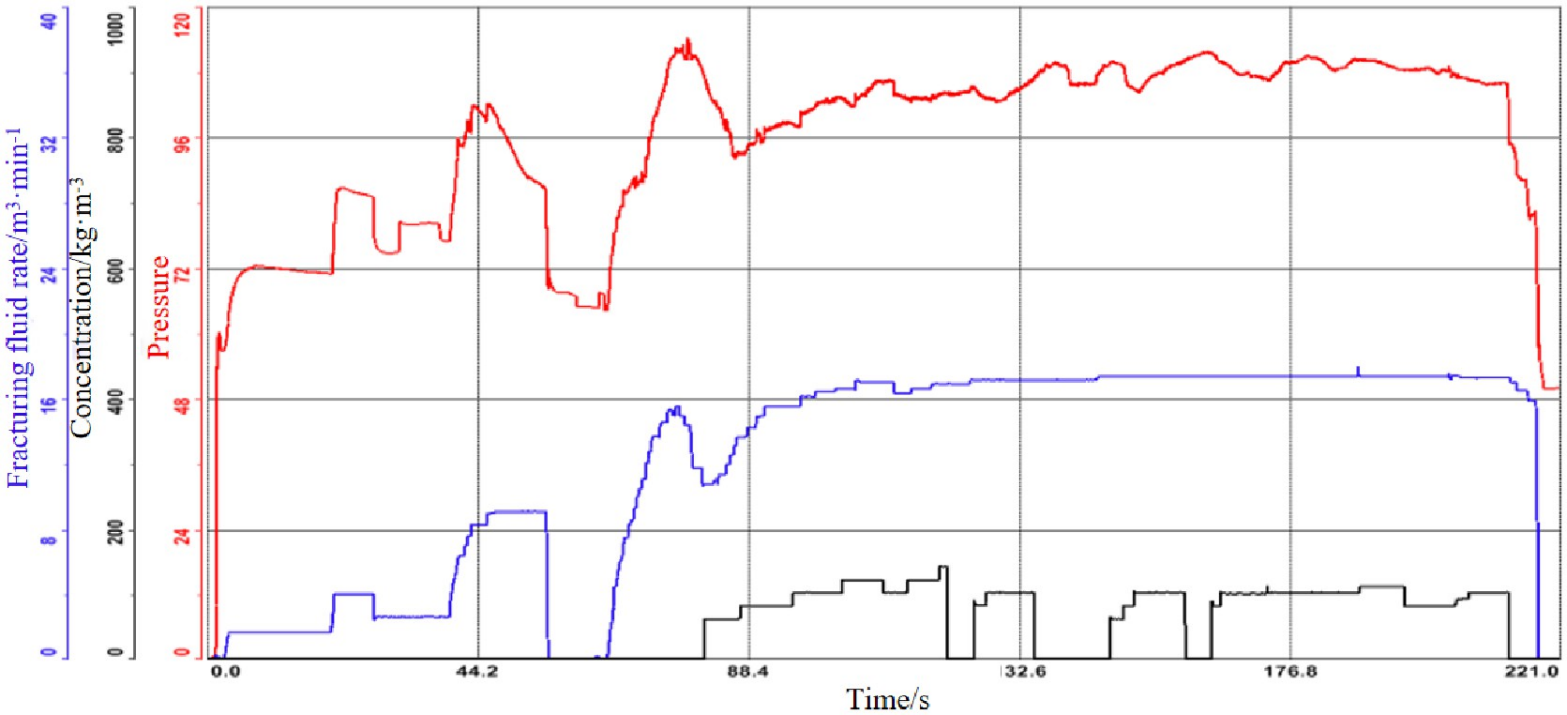
Sand strength 2.94t/m.

Pre-fluid phase of well E: step lift rate is adopted. Quartz sand stage: continuously add sand, slowly increase the displacement, and adopt 20mPa·s in the later stage to carry the quartz sand farther. Ceramide stage: continuously add sand, the main body concentration is controlled within 160kg/m³, and the limit displacement is adopted with low stick and slip water. Figs 12 and 13 showed the fracturing operation curve under different sand intensities.

27 stages of well E have been fractured. The construction displacement is 14~18m³/min and the construction pressure is 91~113.1MPa. The average liquid strength is 30.39m³/m, and the sand strength is 3.10t/m, meeting the design requirements, as shown in Table 11.

**Table 11:** Fracturing parameter design of E well.

	Design parameters	Actual parameters
Fracturing stage length	1643m	1800m
Number of stages	27(60.9m)	30(60.0m)
Fracturing fluid rate	18m^3^/min	14~18m^3^/min
Proppant combination	71%Quartz sand+29%Ceramite	71.1%Quartz sand+28.9%Ceramite
Sanding	Body segment3.5t/m, Risk segment2.0t/m、2.5t/m	Body segment3.60t/m, Risk segment2.69t/m、2.53t/m

#### 5.7.3 Evaluation of post-compression effect

**Fig 14:**
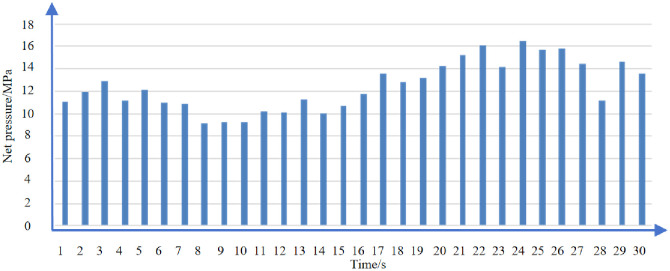
Net pressure of each section of D well.

Fig 14 shows the net pressure in each section of well D. Well D: Net pressure analysis: The average net pressure of the well reaches 12.5MPa, which is greater than the horizontal stress difference of the well 10.2MPa. The fracture complexity is high, and the transformation effect is good.

**Fig 15:**
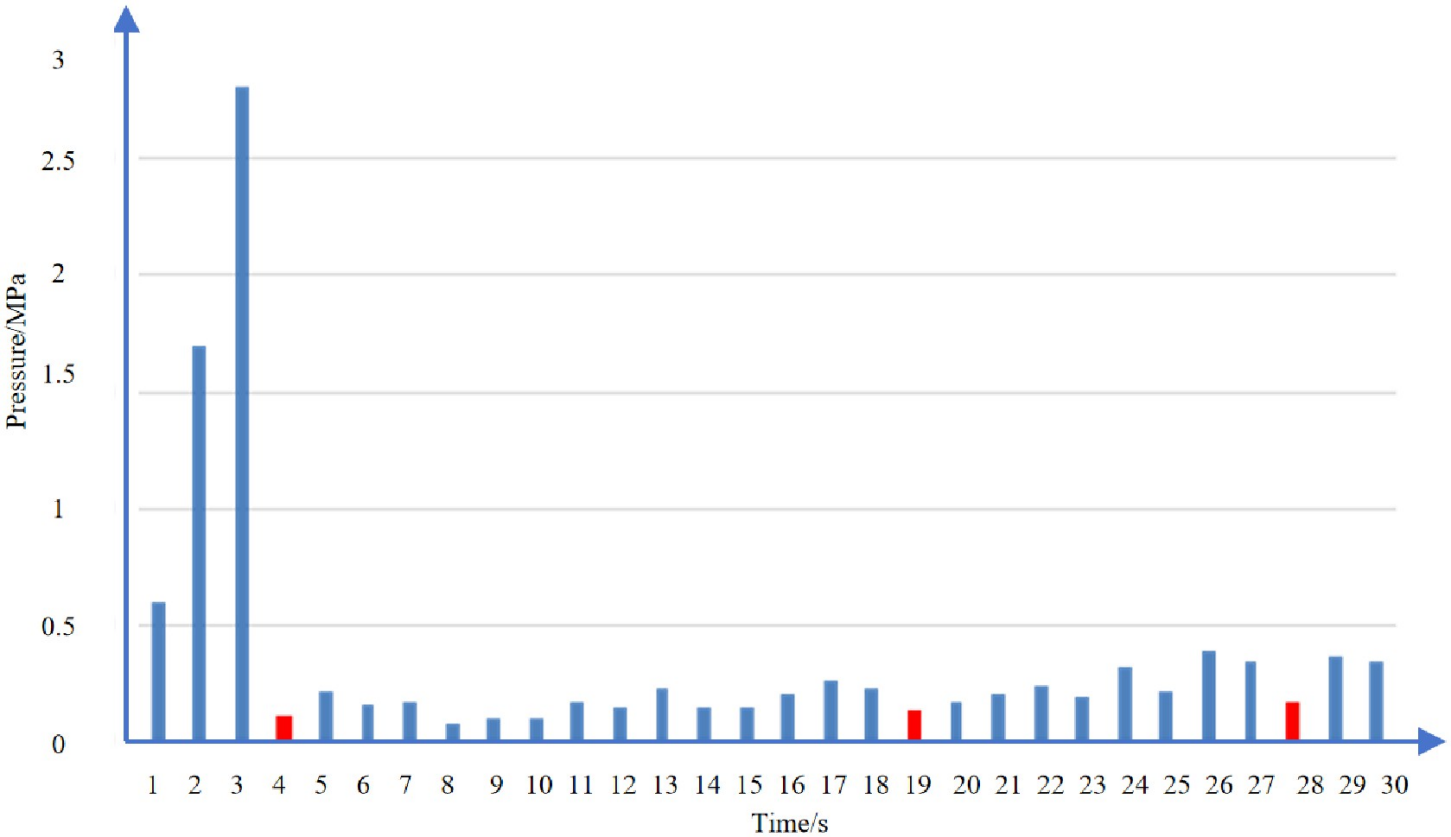
15min pressure drop statistics of each section of D Well (red is natural fracture).

Fig 15 shows the 15min pressure drop statistics of each section of well D. The average pressure drop 15 minutes after pump shutdown in well D is 6.8MPa, and the average 15min pressure drop in the developed natural fracture section is 10.2MPa, which is 82% higher than 5.6MPa in other sections.

**Fig 16:**
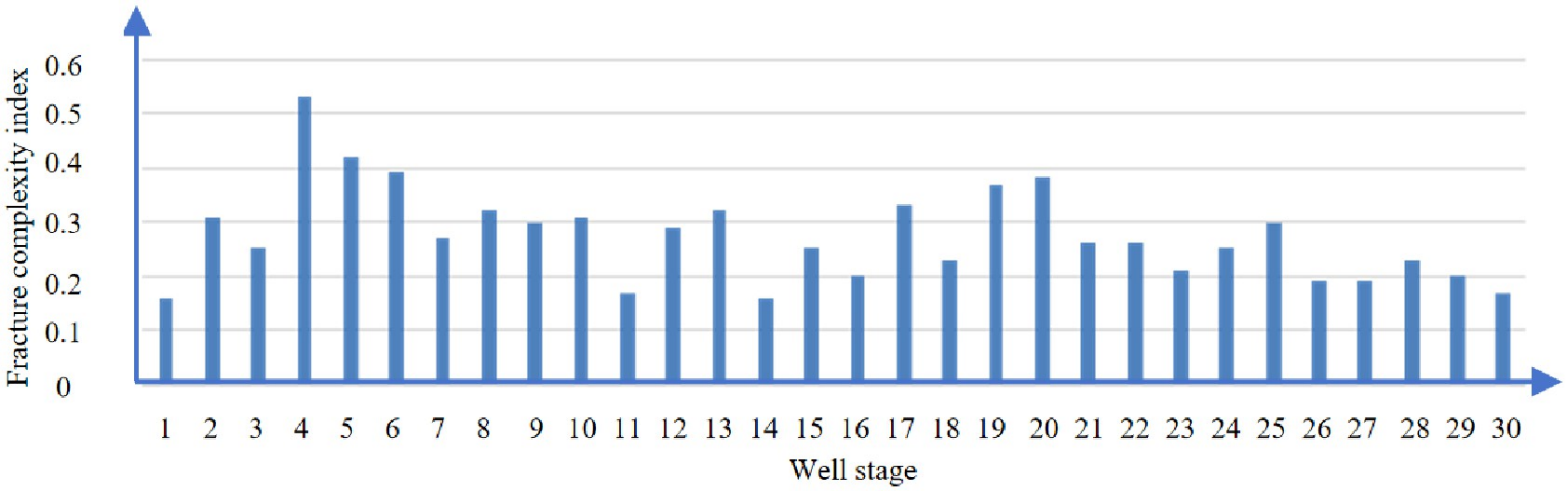
Fracture complex index of D well.

**Fig 17:**
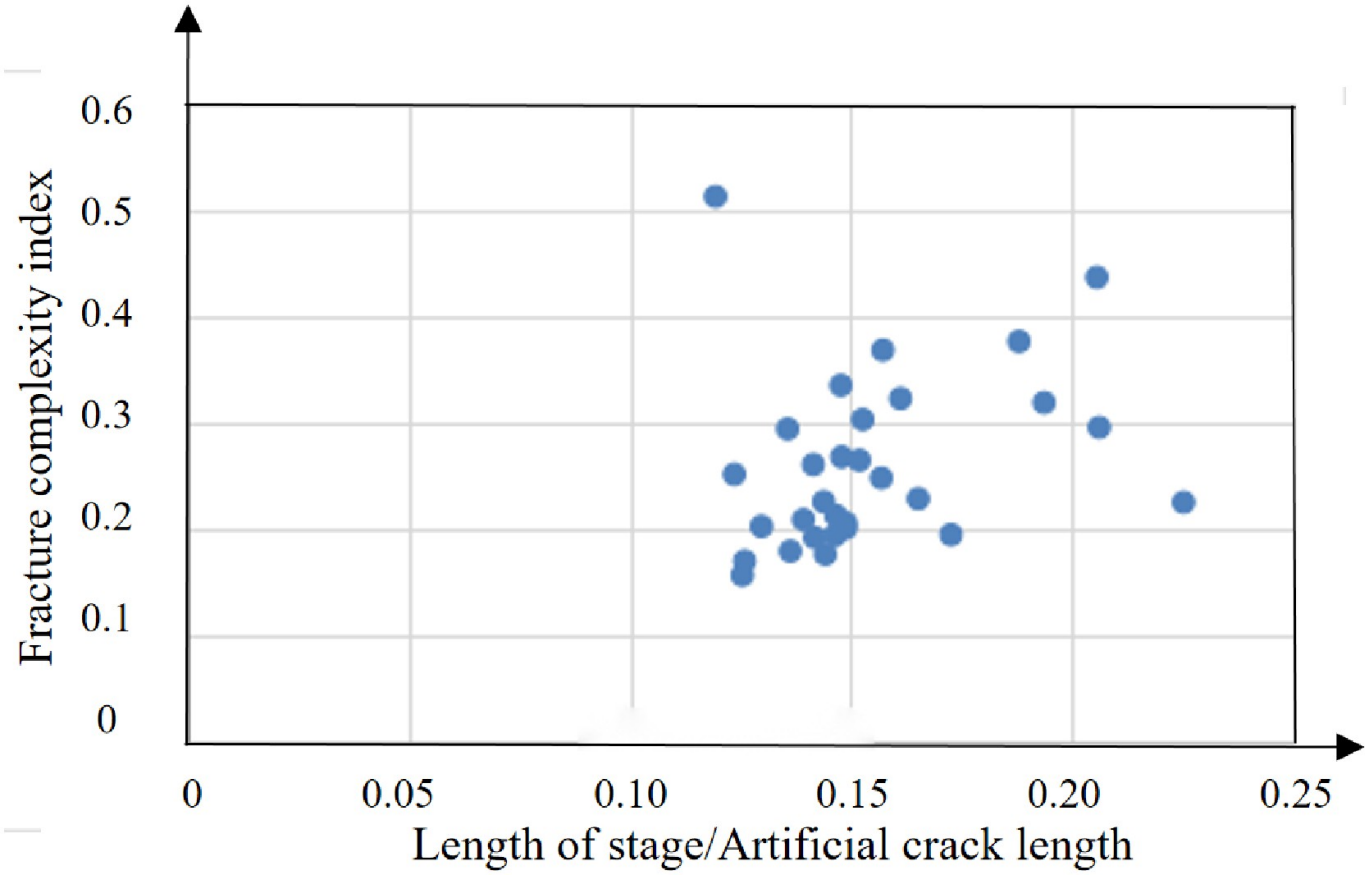
Fracture complex index analysis of D well.

Figs 16 and 17 show the fracture complexity index analysis of well D. The average fracture complexity index of section 1–30 of well D is 0.27.

**Fig 18:**
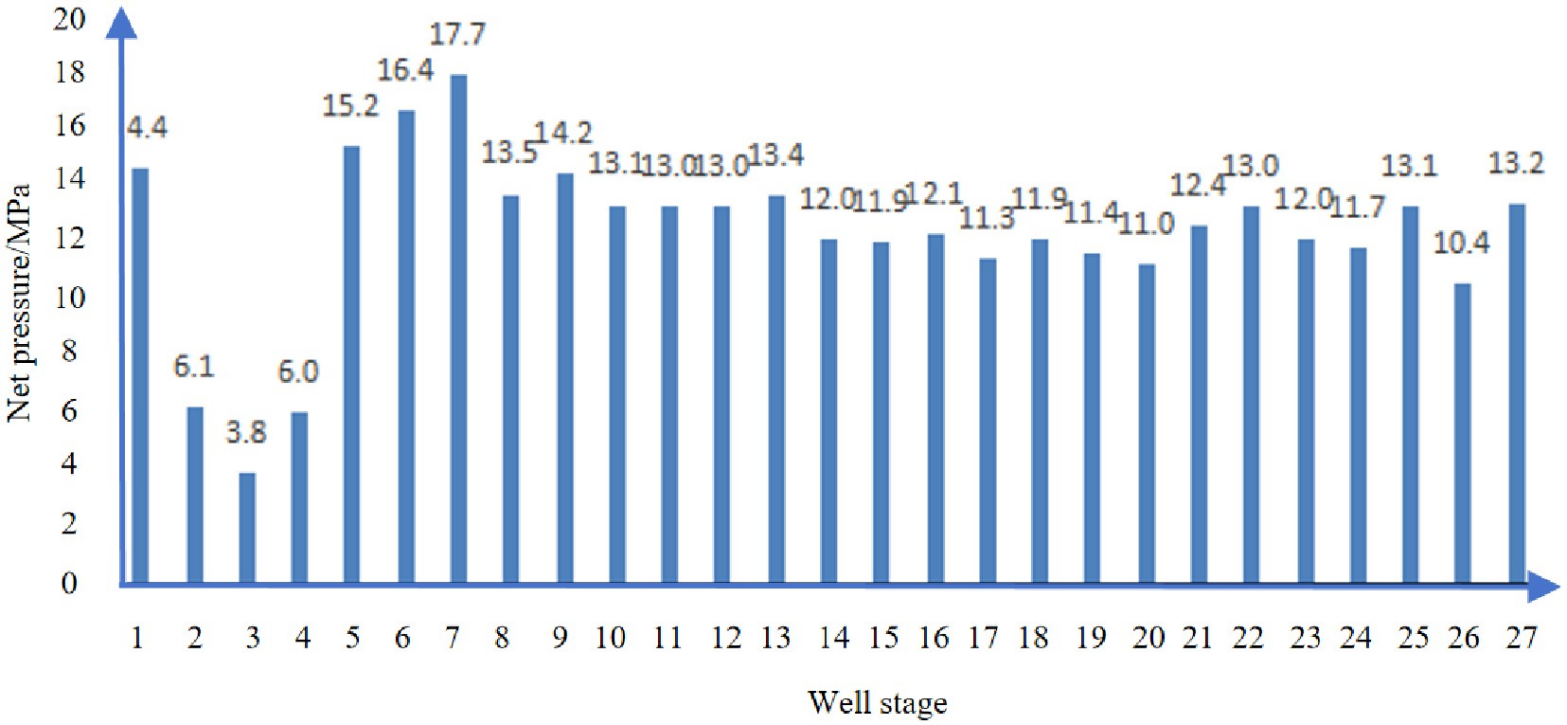
Net pressure of each section of E well.

Fig 18 shows the net pressure in each section of well E. Net pressure analysis of well E: The average net pressure of the well reached 13.0MPa (in which the sensor in section 2–4 was faulty and the pump shutdown pressure was inaccurate), which was greater than the horizontal stress difference of the well 12.1MPa. The fracture complexity was relatively high, and the transformation effect was good.

**Fig 19:**
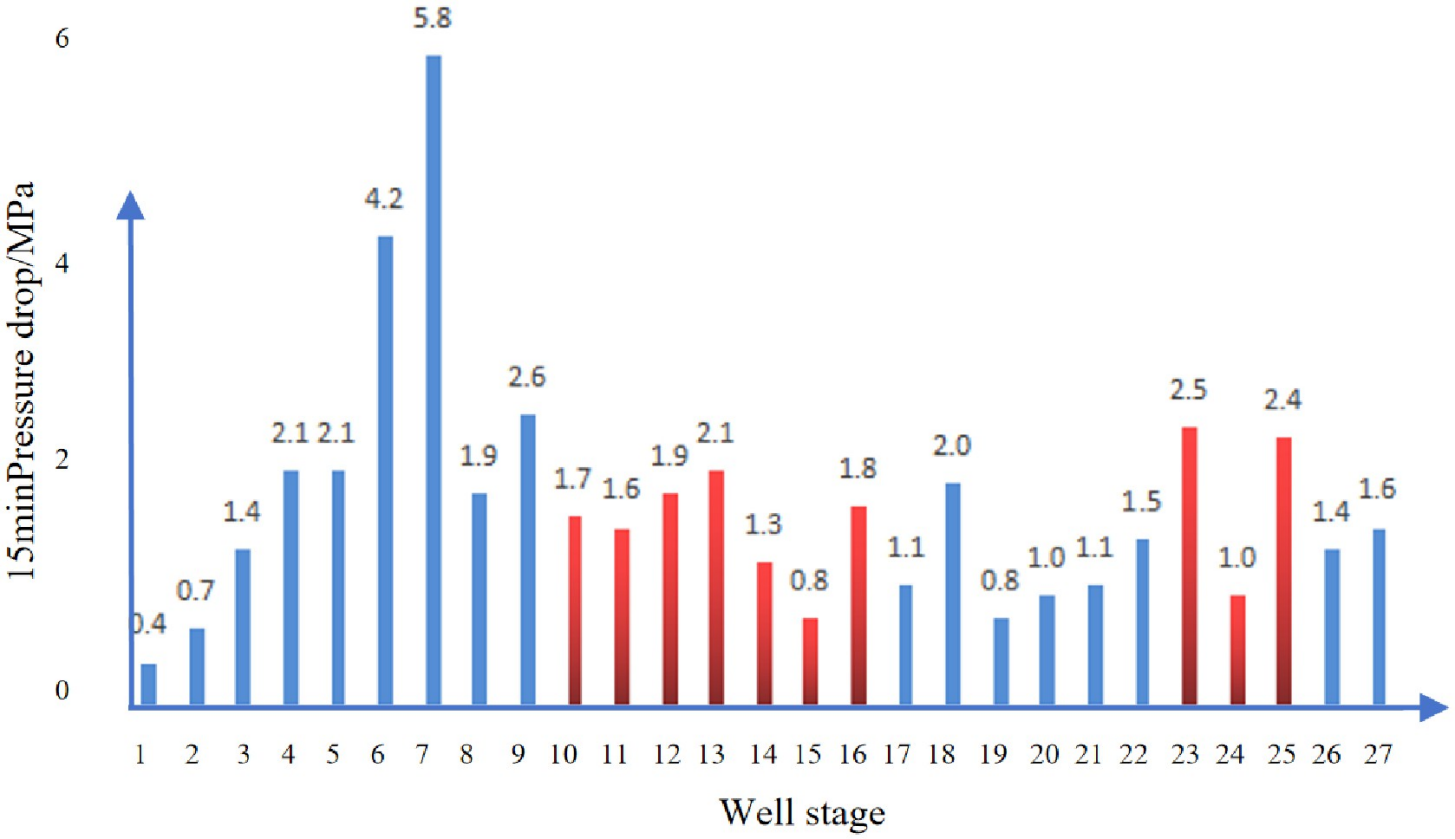
15min pressure drop statistics of each section of E Well (red is natural fracture prediction section).

The average pressure drop in 15 minutes after the well stopped pumping was 1.8MPa, and the average pressure drop in the natural fracture development section was 1.7MPa in 15 minutes, which was not much different from 2.0MPa in the main section. As shown in Fig 19.

**Fig 20:**
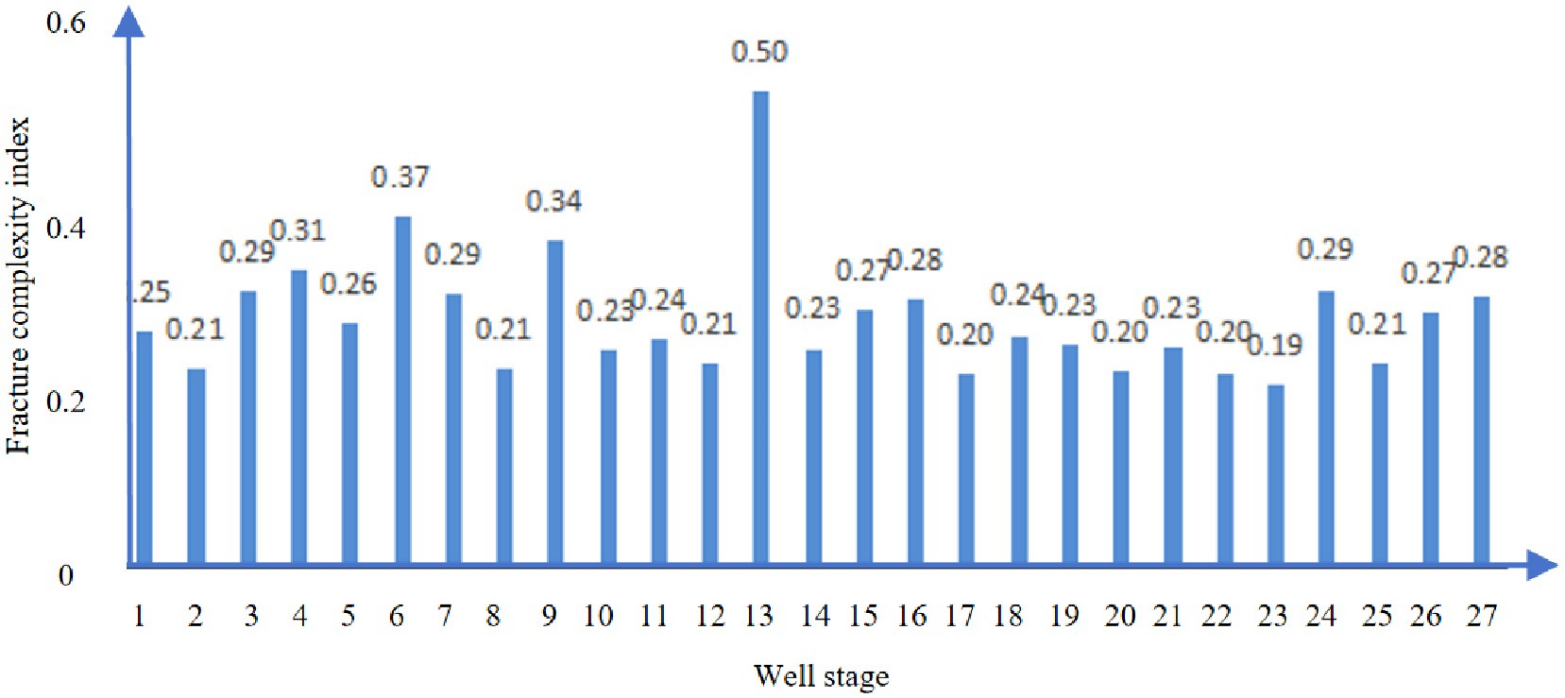
Fracture complex index of E well.

**Fig 21:**
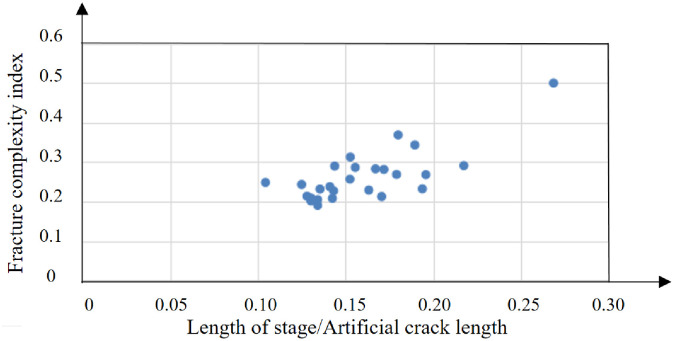
Fracture complex index analysis of E well.

The average fracture complexity index of section 1 to 27 of well E is 0.29, as shown in Figs 20 and 21.

The field results showed that although the depth of well E is larger than that of well D, the number of natural fractures is less, and the horizontal stress difference of well E is larger, the displacement of well E is larger, and the final results show that the fracture complexity index of well E is higher than that of well D, which is consistent with the main control factors affecting the fracture complexity obtained by XSite simulation test.

## 6 Conclusions

Xsite is used to study the formation mechanism and method of complex fractures in shale. Xsite has shown unique advantages in dealing with complex boundary conditions, multiphase flow and interface interaction between fluid and solid. Firstly, the basic principle of Xsite discrete lattice method is introduced. Then, the influence factors of hydraulic fracturing are studied, and the influence of different influencing factors on hydraulic crack morphology, expansion law and crack parameters is analyzed. Then the orthogonal experiments were designed to design the optimal combination of different influencing factors from four aspects: crack shape volume, total crack area, tensile crack area and shear crack area. Field tests were used to verify the test results, The following conclusions are drawn:

The orthogonal test scheme with 6 factors and 5 levels was designed. The influencing factors were set as the number of bedding, injection rate of fracturing fluid, internal friction Angle of bedding, vertical stress difference, high Angle crack inclination and fracturing fluid viscosity. Orthogonal tests show that, a. From the perspective of crack morphology and volume, the optimal fracturing design scheme is as follows: the layer rational number is 5, the bedding friction Angle is 1°, the high Angle crack inclination is 0°, the injection rate is 2m3/min, the viscosity is 20mPa·s, and the vertical stress difference is 10MPa. b. From the perspective of the total crack area, the optimal crack design scheme is as follows: the number of layers is 3, the bedding friction Angle is 60°, the high-angle crack inclination is 30°, the injection rate is 5m3/min, the viscosity is 5mPa·s, and the vertical stress difference is 10MPa. c. From the perspective of tensile crack area, the optimal fracturing design scheme is as follows: the number of layers is 5, the bedding friction Angle is 45°, the high-angle crack inclination is 30°, the injection rate is 3m3/min, the viscosity is 5mPa·s, and the vertical stress difference is 15MPa. d. From the perspective of crack shear area, the optimal crack design scheme is as follows: layer rational number is 3, bedding friction Angle is 60°, high-angle crack inclination is 30°, injection rate is 5m3/min, viscosity is 5mPa·s, and vertical stress difference is 10MPa. The comprehensive consideration of the influence weights of different factors of a variety of quantitative indicators shows that, in this test, the degree of influence on hydraulic crack complexity from large to small is: injection rate, bedding friction Angle, High angle fracture dip angle, viscosity, vertical stress difference and bedding number. The optimal fracturing scheme considering the influence weights of various quantitative criteria is as follows: The layer rational number is 5, the bedding friction Angle is 1°, the high Angle crack inclination is 0°, the injection rate is 2m3/min, the viscosity is 20mPa·s, and the vertical stress difference is 10MPa. b. From the perspective of the total crack area, the optimal crack design scheme is as follows: the number of layers is 5, the bedding friction Angle is 30°, the high-angle crack inclination is 30°, the injection rate is 5m3/min, the viscosity is 5mPa·s, and the vertical stress difference is 10MPa.

Through the field case analysis, we found that even though the depth of well E is greater than that of well D and the horizontal stress difference is larger, the fracture complexity of well E is still higher than that of well D after the high flow rate operation. This observation is consistent with the results of simulation experiments using XSite software. This study provides reference for fracturing scheme design under different engineering background.

## Supporting information

S1 Data Data in the article. (XLSX)
